# Complex Control of GABA(A) Receptor Subunit mRNA Expression: Variation, Covariation, and Genetic Regulation

**DOI:** 10.1371/journal.pone.0034586

**Published:** 2012-04-10

**Authors:** Megan K. Mulligan, Xusheng Wang, Adrienne L. Adler, Khyobeni Mozhui, Lu Lu, Robert W. Williams

**Affiliations:** Department of Anatomy and Neurobiology, University of Tennessee Health Science Center, Memphis, Tennessee, United States of America; Radboud University, The Netherlands

## Abstract

GABA type-A receptors are essential for fast inhibitory neurotransmission and are critical in brain function. Surprisingly, expression of receptor subunits is highly variable among individuals, but the cause and impact of this fluctuation remains unknown. We have studied sources of variation for all 19 receptor subunits using massive expression data sets collected across multiple brain regions and platforms in mice and humans. Expression of *Gabra1*, *Gabra2*, *Gabrb2*, *Gabrb3*, and *Gabrg2* is highly variable and heritable among the large cohort of BXD strains derived from crosses of fully sequenced parents—C57BL/6J and DBA/2J. Genetic control of these subunits is complex and highly dependent on tissue and mRNA region. Remarkably, this high variation is generally not linked to phenotypic differences. The single exception is *Gabrb3*, a locus that is linked to anxiety. We identified upstream genetic loci that influence subunit expression, including three unlinked regions of chromosome 5 that modulate the expression of nine subunits in hippocampus, and that are also associated with multiple phenotypes. Candidate genes within these loci include, *Naaa*, *Nos1*, and *Zkscan1*. We confirmed a high level of coexpression for subunits comprising the major channel—*Gabra1*, *Gabrb2*, and *Gabrg2*—and identified conserved members of this expression network in mice and humans. *Gucy1a3*, *Gucy1b3*, and *Lis1* are novel and conserved associates of multiple subunits that are involved in inhibitory signaling. Finally, proximal and distal regions of the 3′ UTRs of single subunits have remarkably independent expression patterns in both species. However, corresponding regions of different subunits often show congruent genetic control and coexpression (proximal-to-proximal or distal-to-distal), even in the absence of sequence homology. Our findings identify novel sources of variation that modulate subunit expression and highlight the extraordinary capacity of biological networks to buffer 4–100 fold differences in mRNA levels.

## Introduction

There is an extraordinarily high level of variation in expression of messenger RNAs of key inhibitory GABA type-A receptors (GABA(A)R) in human brain. For example, *GABRA1*—a key constituent of the great majority of functional receptors—varies over a 100-fold range in the neocortex of a sample of 187 healthy but elderly humans [1]. In comparison, in the same sample, the *GABRG2* and *GABRB1* receptors “only” vary 10–20 fold. This is a remarkable range that exceeds that which is often achieved in knock-in and knock-down experiments in genetically engineered lines of mice.

This variation in expression is doubly remarkable because dysregulation of GABA(A)R have been linked to a wide range of abnormalities and neurological diseases, including epilepsy, autism, impulsivity, substance abuse disorders, mood, psychiatric disease, and chronic pain. This raises an important question as to the causes and consequences of the high level of endogenous variation among normal humans. Is it a technical artifact of array-based methods? Is it due to difficulties in obtaining high quality RNA from human brain? Or does it reflect flexible use of GABA(A)R subunits to assemble pentameric receptors?

To answer these questions we need to weigh the relative importance of genetic, environmental, and technical sources of variation. What fraction of variation is heritable and what fraction is due to environmental or technical error? What is the functional relevance of these differences at the RNA level? In the large sample of humans studied by Webster and colleagues (2009), each individual has a unique genotype and it is not practical to resample the same genotype many times to estimate or eliminate technical errors. However, these questions can be addressed efficiently using diverse sets of fully inbred strains of mice that model human populations.

We have exploited a large set of inbred strains of mice—the BXD family—to study the expression of GABA(A)R subunits in the brain. This family is composed of both parent strains (C57BL/6J and DBA/2J) and ∼160 lines. While each line is fully inbred, the entire collection is highly diverse and members are segregating for ∼5 million sequence variants. Both parents have been fully sequenced and the progeny have been genotyped at over 7,000 genetic markers. Furthermore, members of this family have been well phenotyped for ∼40 years. This remarkably dense data allows us to define genetic and phenotypic differences between parents and among their progeny. As in humans we detect large differences in subunit expression among members of this family. For example, α2, α4, and β3 subunits vary more than 3-fold even after averaging multiple samples per strain. Here we address four key questions about the molecular genetics of GABA(A)R subunits:

- What are the sources of variation in subunit mRNA expression?
- What genomic regions (QTLs), genes, or even sequence variants contribute to subunit variation?
- To what extent do subunits interact with each other or with other genes critical in synaptic function at the mRNA level?
- What is the functional impact of this variation in terms of behavior and disease susceptibility?

## Results

### Expression, variation, and heritability

We examined expression of GABA(A)R subunits across six brain regions, two species, and multiple expression platforms using a large number of probes and probe sets, and RNA sequencing (RNA-seq). To simplify the presentation much of the analysis is based on expression in the hippocampus and on data generated using the Affymetrix M430 and Exon 1.0 ST platforms (Table S1). Expression values are expressed on a log2 intensity scale. Values of 8 to 9 units correspond to moderate levels of expression (2 to 4 pM), whereas values of 12 to 13 correspond to high levels of expression (∼40 to 80 pM).

A majority of the GABA(A)R subunits are well expressed in the hippocampus. The most abundant subunits include *Gabrb1*, *Gabra5*, *Gabra1*, *Gabrg2*, and *Gabrb2*. Other subunits are more moderately expressed (*Gabra2*, *Gabra3*, *Gabra4*, *Gabrb3*, *Gabrg1*, and *Gabrd*) or expressed at very low levels (*Gabrg3*). Several subunits, including *Gabra6*, *Gabrr1*, *Gabrr2*, *Gabrr3*, *Gabre*, *Gabrp*, and *Gabrq* are not expressed above background in the hippocampus and are not considered in detail further. Extensive data is available for all of these genes and their corresponding probe sets in GeneNetwork (www.genenetwork.org). As shown in Table 1 and Figure S1, there is a good correlation between mean subunit expression on the arrays, gene level expression based on RNA-seq of whole brain, and the intensity of *in situ* expression in the Allen Brain Atlas [2].

**Table 1:** GABA(A)R expression summary.

Symbol	AVG Exon Array/Probe Sets	AVG FC Exon Array	AVG M430 Array/Probe Sets	AVG FC M430 Array	Allen Brain Atlas HIP	RNAseq Gene ID	WB RNAseq AVG FPKM	WB RNAseq BXD FC
*Gabrr3*	7.57/8	4.65	NA	NA	0	NM_001081190	0.00	1.00
*Gabrd*	9.60/11	4.86	9.33/1	2.33	1	NM_008072	13.79	2.93
*Gabrr2*	7.84/10	3.83	(7.5/1)	(1.30)	0	NM_008076	0.06	N/A
*Gabrr1*	7.60/14	4.33	9.68/2	2.04	0	NM_008075	0.42	3.37
*Gabrg1*	8.65/14	6.32	7.97/2	2.19	0	NM_010252	3.51	2.34
*Gabra2*	11.40/18	4.58	10.97/3	4.08	1	NM_008066	10.36	2.79
*Gabra4*	11.22/14	2.95	10.14/2	2.29	4	NM_010251	11.15	2.80
*Gabrb1*	12.07/9	2.57	11.83/2	1.57	3	NM_008069	8.33	1.72
*Gabrg3*	10.26/11	3.29	(7.18/2)	(1.52)	1	NM_008074	3.13	2.78
*Gabra5*	12.03/13	3.34	11.90/1	1.64	5	NM_176942	8.06	2.35
*Gabrb3*	11.53/7	3.41	10.19/1	3.64	5	NM_001038701	5.19	4.74
*Gabrp*	6.94/16	5.49	8.58/1	1.37	0	NM_146017	0.01	N/A
*Gabrg2*	12.55/14	3.07	10.95/2	1.95	3	NM_008073	14.16	3.20
*Gabra1*	12.75/12	2.37	(12.23/4)	(2.13)	4	NM_010250	57.81	2.65
*Gabra6*	6.95/14	38.69	6.74/2	10.44	0	NM_001099641	14.02	N/A
*Gabrb2*	12.22/11	2.81	12.14/1	1.53	4	NM_008070	22.50	2.26
*Gabre*	6.29/14	6.51	7.69/1	1.45	0	NM_017369	0.35	25.93
*Gabra3*	10.20/18	3.83	8.15/2	2.03	2	NM_008067	12.46	1.97
*Gabrq*	6.56/13	5.71	6.78/1	1.45	0	NM_020488	0.73	N/A

HIP = Hippocampus, AVG = Average log2 standardized expression with a mean of 8 and standard deviation of 2, Exon = Exon array platform, M430 = M430 array platform, FC = Fold Change in expression between the highest and lowest strain, () = Measurement includes probes overlapping SNPs as all probe sets are affected, RNAseq = SOLiD platform RNA sequencing, EXPR = Expression. 0 = No expression, 3 = Moderate expression, 5 = High expression in the hippocampal formation. Gene ID = NCBI RefSeq ID, FPKM = read fragments per KB gene model per million sample read fragments.

The expression of a subset of subunits is markedly different among the BXD strains. For example, the expression level of *Gabra1*, *Gabra2*, *Gabra4*, *Gabrb2*, and *Gabrb3* differs by more than 2.5-fold among strains (Table 1). This high variation in mRNA levels is attributable to a combination of technical, environmental, and genetic factors. Some of the variation could be due to hybridization artifacts caused by polymorphisms that overlap probe target sequences [3], [4], [5]. To eliminate signals resulting from this artifact we removed probes overlapping SNPs and small indels (Methods, Table S1). We are confident that the high level of variation shown in Table 1 is not an artifact. To estimate the influence of genetic versus environmental modulation we calculated the heritability of mRNA expression using the large hippocampal data set (Table S2). For many subunits, heritability ranged from 0.40 to 0.95—demonstrating that genetic factors are a major contributor to variation in expression. This heritable variation enables us to identify genetic loci or so-called expression quantitative trait loci (eQTLs) that control subunit expression using genomic extensions of classical gene mapping methods introduced by Damerval and colleagues [6]. These eQTLs can be classified broadly into two types: *cis* eQTLs and *trans* eQTLs. A *cis* eQTL is caused by sequence variants located within or very close to the gene from which mRNA is transcribed. In contrast, a *trans* eQTL is caused by sequence variants located on a different chromosome or more than 10 Mb distant from the cognate gene on the same chromosome.

### Modulation by *cis* eQTLs

**Figure 1:**
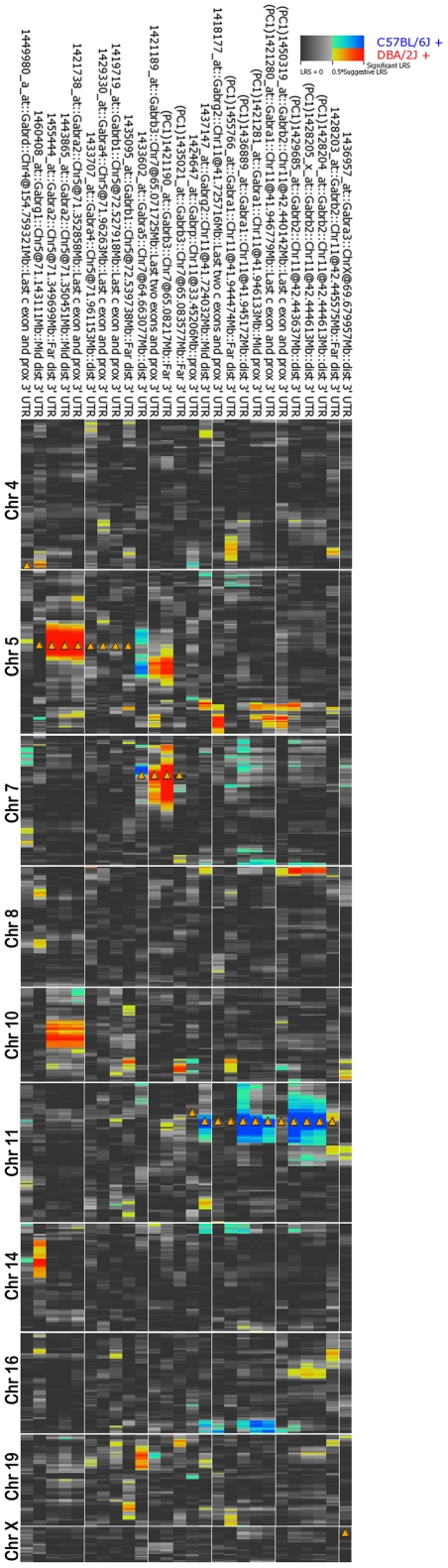
Summary of significant genetic regulation in the hippocampus. Probe sets represent columns and ascending genomic location for each chromosome represent rows. Only chromosomes with a significant QTL (LRS>15) are shown. Several GABA(A)R subunits are regulated by both *cis* and *trans* eQTL (*Gabra1*, *Gabra2*, *Gabrb2*, and *Gabrb3*) whereas some are modified exclusively in *trans* (*Gabra5*, *Gabrg1*). Hue intensity provides an indication of the strength of the association between gene expression and genomic location. Red and blue alleles indicate that the *D* or *B* allele increases trait values, respectively. Arrowheads show the location of each cognate gene. PC1 = first principal component, Chr = Chromosome, Mb = Megabase, Prox = proximal, mid = middle, dist = distal.

In agreement with their high heritability, *Gabra1*, *Gabra2*, *Gabrb2*, *Gabrg2*, and *Gabrb3* are strongly modulated by *cis* eQTLs that have likelihood ratio statistic (LRS) scores above 20 (Figure 1). (An LRS score can be converted to a logarithm of the odds (LOD) score by dividing by 4.61; where LOD is approximately equal to −log(P) of linkage.) Three subunits—*Gabra1*, *Gabrb2*, and *Gabrg2*—are located close together on chromosome (Chr) 11 between 33 and 42 megabases (Mb) and the remaining two subunits, *Gabra2* and *Gabrb3*, are located within GABA(A)R subunit clusters on Chr 5 (near 71 Mb) and Chr 7 (near 65 Mb), respectively. Higher expression of all three genes on Chr 11 is generally associated with the *B* allele inherited from the maternal C57BL/6J strain whereas higher expression of *Gabra2* and *Gabrb3* is always associated with the *D* allele inherited from the DBA/2J paternal strain.

We tested the consistency of the *cis* eQTLs initially detected in hippocampus from the BXD population at four different levels; (1) brain tissue, (2) expression platform, (3) mRNA region, and (4) mouse population.

**Figure 2:**
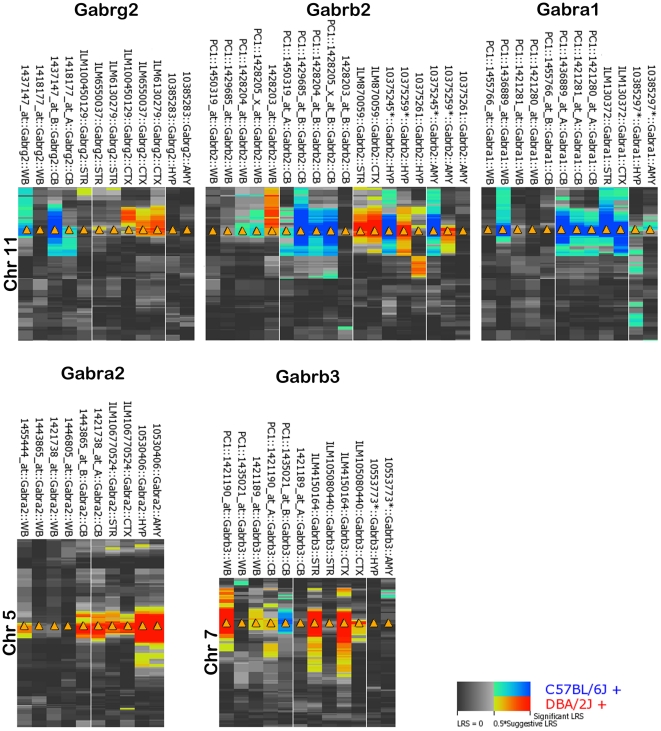
Consistency of *cis* modulation across tissues. Genetic regulation of the 5 subunits regulated by strong *cis* eQTLs in the hippocampus was compared across three different platforms Affymetrix M430 (WB = whole brain, CB = cerebellum), Affymetrix MoGene 1.0 ST (AMY = amgydala and HYP = hypothalamus), and Illumina (CTX = neocortex, STR = striatum). Each probe set was required to have a mean expression of at least 8 in each tissue database. Blue and red indicate an association between the B6 and D2 allele at each locus and higher probe set expression, respectively. The more intense hue indicates a greater association and a corresponding increase in significance. For all data except the HYP and AMY, probes overlapping SNPs were filtered out and a principal component analysis was performed on remaining probes to capture a component (PC1) explaining the majority of the variation in probe set expression. Percent of variance explained by each PC1 ranged from (∼45% to 95%). Because of the large number of probes included for measurement in the HYP and AMY, a probe level analysis was not possible. Some probe sets (indicated by *) contained 1 to 3 probes overlapping a variant. It is hard to predict the overall effect of these variants but, in general, those probe sets with SNPs that have higher expression associated with the B6 allele (blue) have a higher chance of being false due to SNP artifacts. *Cis* modulation of expression across tissues is most consistent for *Gabra2* and *Gabra1*.

Comparison of subunit expression across multiple CNS tissues (whole brain, cerebellum, striatum, cortex, hypothalamus, and amygdala) and several array platforms in the BXD family reveals both common as well as unique genetic control of expression of *Gabra1*, *Gabrb2*, *Gabrb3*, *Gabrg2*, and *Gabra2* (Figure 2). Both *Gabra1* and *Gabra2* have highly conserved *cis*-regulation and high LRS values are always associated with either the *B* allele (*Gabra1*) or *D* allele (*Gabra2*), regardless of brain region or array platform. In contrast, allelic control of *Gabrb2*, *Gabrb3*, and *Gabrg2* is more complex and varies across tissue and platform (Figure 2). Whole brain RNA-seq reveals significant expression differences based on genotype for *Gabra2* (fold change = 1.9, *p* = 0.05) and *Gabra1* (fold change = 19.6, *p* = 0.0002). Higher expression is associated with the *D* allele for both subunits. *Gabra6*, a gene expressed almost exclusively in the cerebellum, is also *cis* modulated (higher expression associated with the *D* allele) (fold change = 4.5, *p* = 0.0004). We detected no significant difference in expression of *Gabrb2*, *Gabrg2*, or *Gabrb3* in our RNA-seq analysis. *Cis*-regulation of *Gabrg2* by local polymorphisms has been confirmed by Ciobanu and colleagues using traditional allele-specific expression assays [4]. For *Gabrg2*, higher expression is associated with the *D* allele (fold change = 1.5, *p* = 4 E-05). Allele-specific expression assays for *Gabra1* failed to confirm a significant expression difference (fold change = 1.03, *p* = 0.09, higher *D* allele expression). Quantitative real time PCR validated the difference in expression for both *Gabra1* (higher expression in the B6 strain) and *Gabra2* (higher expression in the D2 strain) [7].

**Figure 3:**
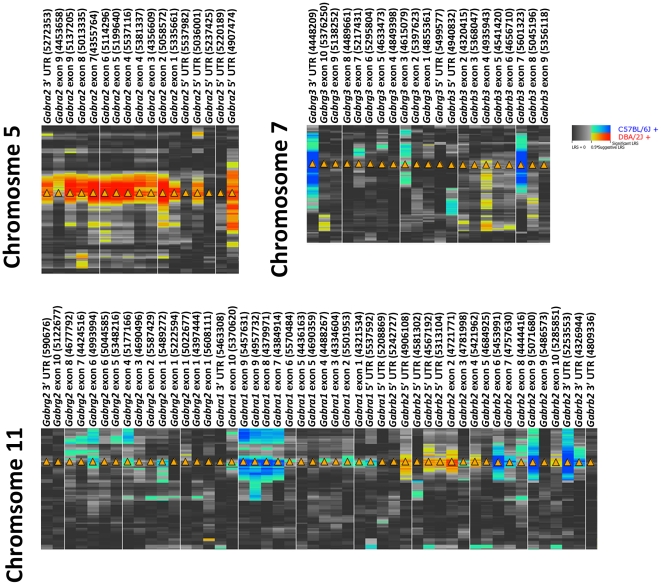
Summary of genetic regulation of GABA(A) R subunits across transcript features. Genetic regulation of exon, intron, and 3′ UTR features for each of the five subunits regulated by strong *cis* eQTLs was measured in the hippocampus using the Affymetrix exon platform (database accession number GN206). Each probe set was required to have a mean expression of at least 10 and all probe sets overlapping SNPs were excluded. Blue and red indicate an association between the B6 and D2 allele at each locus and higher probe set expression, respectively. The more intense hue indicates a greater association and a corresponding increase in significance. Only *Gabra2* shows strong consistency of genetic regulation across mRNA regions.

We analyzed *cis*-regulation of expression at the level of mRNA region using probe sets designed to target distinct transcript regions—5′ UTRs, coding exons, and 3′ UTRs. *Gabra2* has robust and consistent *cis*-modulation across all transcript regions and higher expression is always associated with the *D* allele (Figure 3). In contrast, *cis*-modulation of *Gabra1* and *Gabrg2* expression tends to be associated with high *B* allele expression but this regulation is not congruent across all parts of the transcript. The genetic modulation of *Gabrb2* and *Gabrb3* expression is even more complex. Proximal 5′ features of the *Gabrb2* transcript show opposite allelic regulation compared to distal 3′ features, and only one exon shows strong genetic *cis* modulation for *Gabrb3*. Coexpression between exon probe sets for individual subunits hints at alternative exon usage for *Gabra1* and may provide evidence of multiple isoforms of *Gabrb2* and *Gabrb3* (Table S3) in the hippocampus.

As shown in Table 2, *Gabra1*, *Gabrb2*, and *Gabrg2* are strongly *cis*-modulated in both the BXD family and one other genetic cross. *Gabrb3* and *Gabra2* are regulated by a strong *cis e*QTL in the BXD population and two other crosses.

**Table 2:** Summary of GABA(A)R subunit *cis* modulation among several genetic crosses.

		C57BL/6J by DBA/2J (GN110)	C57BL/6J by C3H/HeJ (GN166)	Cast/EiJ by C57BL/6J F2 (GN171)	LXS (GN133)
***Gabra1***	LRS	68.7	N.S.	21.38	N.S.
	Allele	*B6*	N.S.	*B6*	N.S.
	Marker	rs3693664	N.S.	rs3690160	N.S.
	Location	Chr11 @ 42.18 Mb	N.S.	Chr11 @ 36.83 Mb	N.S.
	Probe set	1436889_at*	10024407236	10023853271	ILM130372
***Gabrg2***	LRS	38.24	17.9	N.S.	N.S.
	Allele	*B6*	*C3H*	N.S.	N.S.
	Marker	rs3693664	rs3703534	N.S.	N.S.
	Location	Chr11 @42.18 Mb	Chr11 @ 32.01 Mb	N.S.	N.S.
	Probe set	1437147_at	10024394157	10023851905	ILM6550037
***Gabrb2***	LRS	79.6	N.S.	315.092	N.S.
	Allele	*B6*	N.S.	*Cast*	N.S.
	Marker	rs3693664	N.S.	rs3693664	N.S.
	Location	Chr11 @ 42.18 Mb	N.S.	Chr11 @ 42.18 Mb	N.S.
	Probe set	1429685_at*	10024404255	10028675696**	ILM100780086
***Gabrb3***	LRS	82.5	N.S.	27.74	123.71
	Allele	*D2*	N.S.	*Cast*	*ISS*
	Marker	gnf07.050.858	N.S.	mCV23672419	rs8269265
	Location	Chr7 @ 63.78 Mb	N.S.	Chr7 @ 64.83 Mb	Chr7 @ 65.05 Mb
	Probe set	1421190_at	10024393218	10018192374	ILM4150164
***Gabra2***	LRS	33.66	92.17	442.306	N.S.
	Allele	*D2*	*C3H*	*Cast*	N.S.
	Marker	rs13478320	rs3672514	rs3681370	N.S.
	Location	Chr5 @ 71.13 Mb	Chr5 @ 74.3 Mb	Chr5 @ 70.53 Mb	N.S.
	Probe set	1455444_at	10024403803	10018193088	ILM3290167

Chr = Chromosome, Mb = Megabase, N/S = Not significant or suggestive. Shading = Possible SNP artifact as the probe overlaps a SNP between parental strains (rs28224563),

* = Probes overlapping SNPs removed and mapping performed with the first principal component of the remaining probes,

** = SNP between parental strains (rs28218735) overlapping probe but the allele effect (*Cast* high) indicates no effect on probe binding. NOTE: ILM4150164 is overlapping rs37397520 which is a SNP between B6 and PWK/PhJ but is probably not segregating in the LXS strains which are derived from A, AKR, BALB/c, C3H/2, C57BL, DBA/2, IS/Bi, and RIII. However the genotype of IS/Bi and RIII is unknown and IS/Bi is now extinct. For *Gabra1*, a probe from probe set rs28224563 overlaps a SNP between parental strains.

### Downstream consequences of genetic variation on higher order phenotypes

GABA(A)R subunits that contain strong polymorphisms controlling their own expression may have multiple downstream effects on the expression of other genes or on higher order functional and behavioral traits. These effects should in turn map back to the location of the gene and the causal sequence variant. For example Li, Mulligan, and colleagues used this approach to extract a large number of phenotypes controlled by a *B* vs *D* polymorphism in catechol-O-methyltransferase (*Comt*) [8]. We find that anxiety-associated phenotypes are associated with variants near *Gabrb3* (LRS>12, *B* allele) on Chr 7 (GeneNetwork BXD Trait IDs: 11389, 11390, 11385, 11388) [9]. Despite high endogenous variation in expression, no phenotypes map back to the locations of *Gabra1*, *Gabrb2*, and *Gabrg2* on Chr 11 or *Gabra2* on Chr 5.

### Trans modulation of individual subunits

We identified five subunits with hippocampal expression variation associated with unique *trans* eQTLs that have LRS scores above 15 (Figure 1 and Table S2). Within each of these five *trans* eQTL we nominated candidate genes that may control subunit expression based on five criteria: (1) correlation with GABA(A)R subunit expression in the hippocampus, (2) overlapping expression patterns in the hippocampal formation (Allen Brain Atlas), (3) true *cis* modulation of candidate regulatory genes in the hippocampus, (4) partial correlation analysis, and (5) significant correlation in normal aged human brain (database accession number GN314) [10]. These criteria were selected based on previous studies, principals of QTL mapping, and translational relevance (see methods).

#### *Gabra1*

Collectively, *Gabra1* probe sets target the last coding exon and proximal 3′ UTR (1421280_at), the proximal to middle portions of the 3′ UTR (1421281_at), and the distal segment of the 3′ UTR (and 1436889_at). Probe sets 1421280_at and 1421281_at are modulated by *trans* eQTLs on Chr 16 at 92.79 Mb (LRS = 18.4, higher expression associated with the *B* allele) and 97 Mb (LRS = 17.7, higher expression associated with the *B* allele), respectively. No candidates were identified near 97 Mb. The best candidate within the *trans* QTL region at 92.79 Mb is Son cell proliferation protein (*Son*). Two probe sets of *Son* (1420951_a_at and 1439074_a_at) are correlated with *Gabra1* probe set 1421280_at (*r*>0.39) and retain the highest partial correlation. *Son* is polymorphic between *B* and *D* haplotypes and is highly expressed in hippocampus. In human brain, *SON* (201086_x_at) is highly correlated (*r* = 0.56) with expression of the middle and distal region of *GABRA1* (244118_at) but not the last two exons and proximal 3′ UTR.

#### *Gabra2*

A region on Chr 10 centered on ∼86 Mb contains genes and variants that influence *Gabra2* expression in the hippocampus (LRS>15, higher expression associated with the *D* allele). Within this chromosomal region there are several candidates whose expression is strongly *cis*-regulated, correlated with *Gabra2* expression, and that share the pattern of expression of *Gabra2* in the hippocampus. Candidates include host cell factor C2 (*Hcfc2*, 1438328_at, 82.20 Mb, LRS = 107.6), 5′ nucleotidase domain containing 3 (*Nt5dc3*, 1443558_s_at, 86.24 Mb, LRS = 69.189), synapsin 3 (*Syn3*, 1443006_at, 85.66 Mb, LRS = 25.078), and stabilin 2 (*Stab2*, 1419423_at, 86.3 Mb, LRS = 55.707). Expression of *Nt5dc3*, *Syn3*, and *Hcfc2* is negatively correlated with *Gabra2* (*r* = −0.52, −0.35, and −0.43, respectively) while *Stab2* expression is positively correlated (*r* = 0.39). *Nt5dc3* and *Hcfc2* have moderate to high expression in hippocampal regions CA1 through CA3, as well as in the dentate gyrus. *Syn3* is relatively abundant in dentate gyrus, whereas *Stab2* is not abundantly expressed. The highest partial correlation after correction was with *Nt5dc3*. Of the three candidates, *Hcfc2* (235264_at) has the highest correlation with *GABRA2* (1554308_s_at) expression in human brain (*r* = 0.63).

#### *Gabra5*

Expression of *Gabra5* is significantly regulated by sequence variants on Chr 19 around 16 Mb (LRS = 17, higher expression associated with the *D* allele). The strongest candidate is riboflavin kinase (*Rfk*, 17.4 Mb). Expression of *Rfk* (1415737_at) varies 2-fold across BXD strains, is positively correlated (*r* = 0.431) with *Gabra5* probe set expression, and is well expressed in the hippocampus. *Rfk* is also highly polymorphic and expression is associated with a strong *cis* eQTL (LRS>50) with ∼50% higher expression from the *D* allele. A ∼3-fold variation between the B6 and D2 strains is also confirmed by RNA-seq. In humans, expression of *RFK* (203225_s_at) is strongly correlated (*r* = 0.56) with *GABRA5* (206456_at) expression in several brain tissues from normal aged brain.

#### *Gabrb2*

*Gabrb2* probe sets collectively target different mRNA features. Expression of probe sets targeting the middle to distal segment of the 3′ UTR are modulated in *trans* by a region on Chr 8 at ∼14.5 Mb. Modulation is significant for some probe sets (1429685_at, LRS = 16.3) and suggestive for others (1428205_x_at and 1428204_at, LRS>10) and always associated with higher expression from the *D* allele. The best candidate gene is kelch repeat and BTB (POZ) domain containing 11 (*Kbtbd11*, 1430073_at, 15.0 Mb). *Kbtbd11* is polymorphic, has high expression in the hippocampus, and is correlated with the expression of *Gabrb2* (*r* = 0.41). The expression of *Kbtbd11* is also modulated by a strong *cis* eQTL (LRS = 110.8, 14.48 Mb). In human brain, *KBTBD11* (204301_at) expression is strongly negatively correlated with the expression of multiple *GABRB2* probe sets targeting the last exon and proximal 3′ UTR (207352_s_at, *r* = −0.53) to the distal 3′ UTR (1557122_s_at, *r* = −0.69). The function of this gene is unknown.

#### *Gabrg1*

A significant *trans* eQTL on Chr 14 at ∼60 Mb modulates expression of *Gabrg1*. Candidate genes include, *Atp8a2* (probe set 1440627_at, mean = 8.83, *r* = −0.533, LRS = 26.9 at 59.82 Mb), *Ebpl* (1417298_at, mean = 9.99, *r* = 0.45, LRS = 26 at 61.92 Mb), and *Tnfrsf19* (1448147_at and 1415921_a_at, mean = 11, *r* = 0.45, LRS = 17.3 at 59.83 Mb). *In situ* binding intensity in the hippocampus is low for *Atp8a2*, moderate for *Tnfrsf19*, and high for *Ebpl*. All three remain correlated with *Gabrg1* after control for linkage disequilibrium, but *Atp8a2* and *Tnfrsf19* retain the highest partial correlation. All three are polymorphic between *B* and *D* haplotypes and there is evidence of differential isoform expression in RNA-seq data sets for both *Atp8a2* and *Tnfrsf19* (see ucscbrowser.genenetwork.org). Only *EBPL* (223306_at) is modestly correlated (*r* = 0.43) with *GABRG1* (241805_at) expression in human brain, although the function of this gene is not known.

### Subunit coexpression

**Figure 4:**
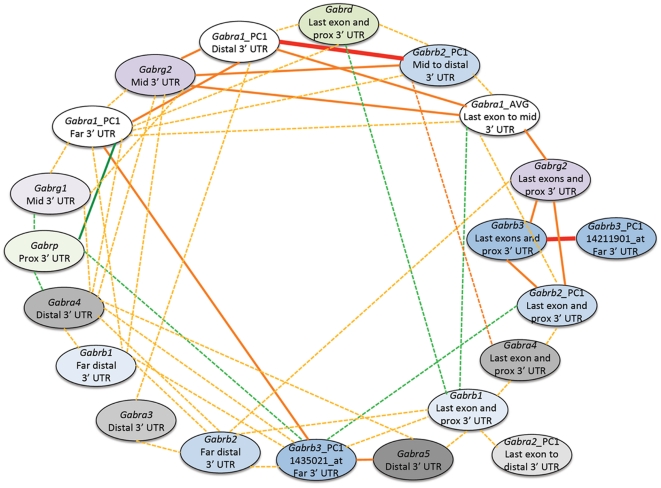
Coexpression of GABA(A) Receptor subunits in the hippocampus. Bold lines indicate a correlation between 1 and 0.7 while normal and dashed lines indicate a correlation between 0.7 and 0.5 and 0.5 and 0.3, respectively. Warm colors represent positive correlations while cool colors represent negative correlations. Probes containing SNPs were removed and a principal component analysis (PCA) was used on the remaining probes to capture the component explaining the majority of the expression variation (PC1) in probe expression among BXD strains. PCA was used to extract PC1 for a single subunit when more than two probe sets were strongly correlated (*r*>0.8) and the average (AVG) was used when two probe sets were strongly correlated. prox = proximal, mid = middle. The highest positive correlations are between subunits targeting the same mRNA region. This is especially true for members of the most common heterotrimeric receptor—*Gabra1*, *Gabrb2*, and *Gabrg2*.

Genetic variation results in profound and highly variable patterns of transcript expression among individuals. We can leverage this covariation to identify potential partnerships among subunits and among members of larger GABA(A)R-associated expression networks. We examined subunit coexpression signatures from BXD hippocampus (database accession number GN110) to detect possible subunit combinations and joint expression of functional GABA(A)R subtypes (Figure 4 and Table S4). As expected, subunits known to form the major heterotrimeric receptor [11], [12]—*Gabra1*, *Gabrb2*, and *Gabrg2*—are generally well and positively coexpressed—higher or lower expression of one isoform in a particular strain being associated with matched variation of functionally associated isoforms.

### Discordant expression

A more intriguing and unexpected finding is that probe sets representing different regions of the same subunit (e.g, last coding exons, proximal 3′ UTRs, and distal 3′ UTRs) are often not correlated and have non-overlapping sets of covariates (Figure 4 and Table S4). *Gabra4*, *Gabrb2*, *Gabrb3*, and *Gabrg2* each have two or more probe sets targeting different parts of the same mRNA that are uncorrelated. These discordant probe sets have excellent sequence specificity, are all well expressed, and have biologically relevant expression correlates. The inconsistency can be resolved by comparing expression patterns between similar regions of different subunits. For example, probe sets of *Gabra1*, *Gabrb2*, and *Gabrg2* that target 3′ coding exons and the proximal 3′ UTR are highly correlated, as are probe sets targeting the middle and distal 3′ UTR. Within these two regions probe sets have an average correlation of 0.50 and 0.65, respectively. Many highly correlated (*r*>0.40) subunit pairs also follow this trend of coexpression within mRNA region: *Gabra2* and *Gabrb1* (last coding exons and proximal 3′ UTR), *Gabra4* and *Gabrg1* (more distal 3′ UTR), *Gabra4* and *Gabrg2* (more distal 3′ UTR), *Gabra5* and *Gabrb3* (more distal 3′ UTR), and *Gabra4* and *Gabra5* (more distal 3′ UTR).

To evaluate the consistency and biological relevance of this finding, we examined coexpression between probe sets targeting different mRNA regions of the same subunit across brain tissues in both mouse and human. We were able to replicate the striking correlation between probe sets targeting different regions of the same mRNA in whole brain and cerebellum (database accession numbers GN123 and GN56, respectively) from the BXD family for *Gabra1*, *Gabra3*, *Gabrb1*, *Gabrb2*, *Gabrg2*, and *Gabrb3* (Table S5). We were also able to replicate the complex correlation pattern between coding exons and different regions of the 3′ UTR for *Gabra5*, *Gabrb2*, *Gabrb3*, and *Gabrg3* in normal adult human brain tissue (database accession number GN314) (Figure 2). This pattern of mRNA regional covariation across probe sets in human varies depending on cortical region.

### RNA-sequencing

To explore GABA(A)R subunit coexpression networks on a more global level, we calculated correlations based on gene-level expression data from whole brain RNA-seq (Figure S3). This analysis corroborates some features of the array data, including the strong coexpression of *Gabra1*, *Gabrb2*, and *Gabrg2*, as well as a strong positive correlation for most of the subunits. Subunits with negative correlations in the RNA-seq coexpression network relative to most other isoforms include *Gabra2*. Similar to the complex correlation pattern between different mRNA regions of GABA(A)R subunits on the M430 array, we also see strong negative correlations between each predicted isoform of *Gabrb3* and *Gabra6*.

### Coordinated genetic regulation of subunit expression and impact on phenotype

Are sets of different subunits and their coexpression networks in the hippocampus controlled jointly by common *trans* eQTLs? We identified three unlinked regions, all on Chr 5 with peaks at ∼90–92 Mb, 117–120 Mb, and 137–138 Mb (Figure S4) that modulate expression of multiple subunits. The most proximal region of Chr 5 controls expression of the last coding exon and the proximal and middle part of the 3′ UTR of *Gabrb3* and the distal 3′ UTR of *Gabra5*. The middle QTL controls the expression of middle to distal parts of the 3′ UTR of four subunits—*Gabra1*, *Gabrb2*, *Gabrb3*, and *Gabrg2*. The last QTL modulates the expression of the last coding exon and the proximal 3′ UTR of six subunits—*Gabra1*, *Gabra2*, *Gabra3*, *Gabrb2*, *Gabrb3*, and *Gabrg2*. The last coding exons and proximal 3′ UTR of four of these subunits (*Gabra1*, *Gabrb2*, *Gabrb3*, and *Gabrg2*) are jointly—albeit unequally—regulated by the middle and distal Chr 5 loci (Figure S4). Higher expression is associated with the *D* allele at these three loci with the exception of the distal 3′ UTR of *Gabra5* and *Gabrb3*, and the middle 3′ UTR of *Gabrg1*. We identified candidate genes in each interval that may regulate the network covariation based on criteria described previously: (1) correlation with GABA(A)R subunit expression in the hippocampus (accession number GN110), (2) overlapping expression patterns in the hippocampal formation (Allen Brain Atlas), (3) true *cis* modulation of candidate regulatory genes in the hippocampus, (4) partial correlation analysis.

- The proximal QTL contains three candidate genes modulated by strong *cis* eQTLs—*Anxa3* (1460330_at, 97.3 Mb, LRS = 43.8), *Naaa* (1452067_at, 92.7 Mb, LRS = 87.5), and *Cox18* (1415710_at, 90.64 Mb, LRS = 148.8). The *B* allele at this locus is associated with increased expression of *Anxa3* and *Naaa* and the *D* allele is associated with higher expression of *Cox18*. Only *Naaa* has moderate to high *in situ* labeling in the hippocampus. *Gabra5* expression is correlated with *Anxa3* (*r* = 0.43), *Naaa* (*r* = 0.58), and *Cox18* (*r* = −0.26). Expression of the *Gabrb3* probe set representing the middle portion of the 3′ UTR is also correlated with *Anxa3* (*r* = −0.31), *Naaa* (*r* = −0.27), and *Cox18* (*r* = 0.44). The highest partial correlation is between *Naaa* (N-acylethanolamine acid amidase) and both *Gabra5* and *Gabrb3*.
- The middle QTL contains three genes controlled by strong *cis* eQTLs—*Nos1* (1438483_at, LRS = 31.1 at 118.33 Mb), *Rasal1* (1458643_at, LRS = 31.7 at 119.76 Mb), and *Thrap2* (1434602_at, LRS = 100 at 118.40 Mb). Probe sets for each candidate are correlated (|*r*|>0.3) with multiple GABA(A)R probe sets. Intensity of *in situ* label in hippocampus is high for all three.
- The distal QTL, identified previously as *Trans5a*, modulates the expression of multiple subunits and also regulates the expression of hundreds of other transcripts in the hippocampus [13]. Overall and colleagues nominated *Zkscan1* as a candidate *trans* regulatory gene in this region. We re-examined *Trans5a* with specific reference to GABA(A)R subunits. This region contains three genes associated with strong *cis* eQTLs that contain multiple sequence polymorphisms and are involved in transcriptional processes—*Taf6*, *Zipro1*, and *Zkscan1*. Both *Taf6* and *Zkscan1* are abundantly expressed in the hippocampal formation but *Taf6* is not as well correlated with subunit expression. Both *Zipro* and *Zkscan1* are correlated (|*r*|>0.5) with the expression of one or more GABA(A)R subunits but *Zipro1* expression is most correlated with *Gabrg1* expression, only. In contrast, *Zkscan1* (1447944_at) expression is highly correlated (*r*>0.5) with that of multiple subunits (last exon and proximal 3′ UTR probe sets of *Gabrb3*, *Gabrg2*, *Gabra1*, and *Gabrb2*). *Zkscan1* probe set 1447944_at also retained the highest partial correlation with GABA(A)R subunits, indicating a residual biological signature that preserves the structure of the network.

Gene variants within the middle and distal regions (118–120.5 Mb, and 137–138 Mb) modulate the expression of multiple GABA(A)R subunits and have a large impact on the expression of synaptic genes in the hippocampus. Variation within this region would be expected to have downstream effects on biological function. Multiple phenotypes are modestly to highly associated with genetic variation on Chr 5 including, doublecortin staining in the hippocampus (a measure of adult neurogenesis), behavioral response to cocaine injection, locomotor response to paraoxon injection, climbing scores after methamphetamine injection, locomotor response after saline injection, and multiple anxiety measures (Table S6).

### Conserved coexpression in mice and humans

A survey in GeneNetwork across human and mouse data sets generated using different tissues and array platforms (see methods) identified a novel subset of genes that covary (*r*>0.30) with six or more GABA(A)R subunits. These include *Gucy1a3* and *Gucy1b3* (Table S7) that interact functionally to form a soluble guanylate cyclase heterodimer involved in GDP/GTP balance and nitric oxide-mediated signal transduction [14]. Another example is platelet-activating factor acetylhydrolase 1B sububit alpha (*Pafah1b1*). Also known as *Lis1*, this gene is a key regulator of neuronal signal transduction and the cytoskeleton, specifically through the rho GTPase *Cdc42* (Table S8) [15].

**Figure 5:**
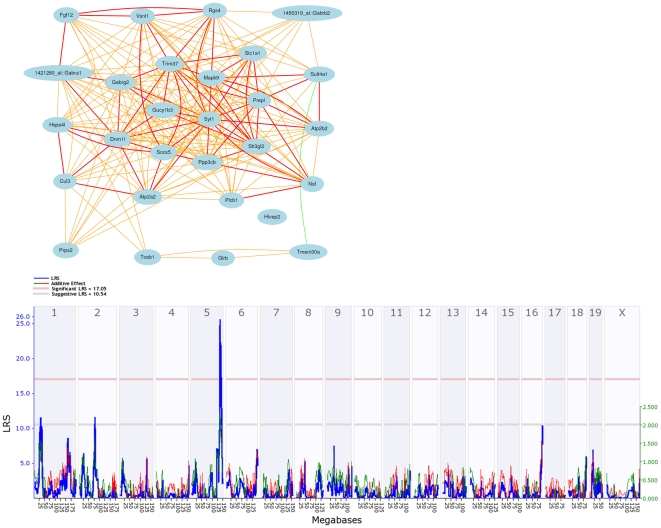
Conserved members of the GABA(A)R gene network. (A) *Gabra1*, *Gabrb2*, and *Gabrg3* are generally well coexpressed across species and platforms. The set of highly conserved correlates include many synaptic genes such as *Syt1* and *Sh3gl2*. Bold lines indicate a correlation between 1 and 0.7 while normal and dashed lines indicate a correlation between 0.7 and 0.5 and 0.5 and 0.3, respectively. Warm colors represent positive correlations while cool colors represent negative correlations. (B) Expression of conserved module members is regulated by *Trans5a*. LRS score and additive effect are shown on the Y axis in blue and green, respectively. The blue line plots the strength of the association between gene expression and chromosomal location. The genome wide significant and suggestive threshold is shown by the red and grey horizontal line, respectively. Red and green lines show the effect of the D or B allele on trait values, respectively. The upper X-axis shows location by chromosome and the lower X-axis shows location in megabases.

To extend the family of novel and conserved genes associated with GABA(A) receptor networks we mined a massive (1,000 samples) and well integrated data set including human and mouse brain tissue from various individuals and expression platforms [16]. Subunits that comprise the major heterotrimeric receptor subtype—*Gabra1*, *Gabrb2*, and *Gabrg2*—are consistently and highly coexpressed compared to other subunits. The conserved set of correlates for these subunits includes *Syt1*, *Mapk9*, *Rgs4*, and *Sh3gl2* (Figure 5A). Strikingly, the core conserved module members from Miller and colleagues [16] also have highly correlated expression in the hippocampus of the BXD family (database accession number GN110) and are regulated by the *Trans5A* locus (Chr 5 at ∼133 to 140 Mb) (Figure 5B). We used WebGestalt [17] to evaluate whether module members are enriched for specific functions. This gene set is enriched (adjusted *P*<0.05) for signal transduction (*Hivep2*, *Atp2a2*, *Gabrg2*, *Rgs4*, *Plcb1*, *Socs5*, *Fgf12*, *Mapk9*, *Glrb*), synaptic transmission (*Atp2b2, Glrb, Syt1, Gabrg2*), ribonucleic biosynthetic process (*Atp2b2, Prps2, Atp2a2*), proteolysis involved in cellular catabolic process (*Socs5, Tceb1, Cul3, Trim37*), and the synapse (*Glrb, Syt1, Gabrg2*).

## Discussion

### Expression variation and local modulation of subunit expression

Many GABA(A)R subunits, including, *Gabra1*, *Gabra2*, *Gabra5*, *Gabrb2*, *Gabrb3*, and *Gabrg2*, have remarkably high levels of variation in expression among both inbred strains of mice and normal human populations [1]. Sources of variation include genetic, environmental/epigenetic, and technical factors. We have controlled for several major technical sources of variation, including hybridization artifacts generated by SNPs and small insertions and deletions [4]. Environmental variation within colonies of laboratory mice is modest. After the reduction of many non-genetic sources of variance, there are still large differences that are clearly due to downstream effects of sequence variants—either *cis* effects in or near GABA(A)R genes, or *trans* effects mediated by distant variants and gene loci.

Compared to genetic modulation of expression by *trans* eQTLs, modulation by *cis* eQTLs is generally much stronger and more consistent across platforms, tissues, and mRNA regions. In general recombination that occurs within a gene is exceedingly rare and most genes and gene clusters are inherited as a block from either the B6 or D2 parent. Highly consistent expression would then be expected across mRNA region and tissues when a single mRNA product is produced from a single gene loci inherited in this fashion. For example, most regions of *Gabra2* mRNA are consistently *cis* modulated with low expression associated with the *B* allele regardless of platform, tissue, or cross (Figure 1, Figure 2, and Figure 3). Remarkably, the widely used C57BL/6J parent strain expresses by far the lowest level of *Gabra2* mRNA among 99 inbred strains we have studied [13]. This locus is largely identical by descent among the parental strains and the only *B* vs. *D* sequence variants near *Gabra2*—eight small indels and nine SNPs—fall within introns or intergenic regions (ucscbrowser.genenetwork.org). One or more of these local variants contribute to a 2-to 3-fold difference in *Gabra2* expression.

In contrast and contrary to our expectations, *cis* modulation of other subunits is not consistent across platforms, tissues, and mRNA/probe set target regions (Figure 1, Figure 2, Figure 3, and Table S3) even though these genes and gene clusters are inherited as a single haplotype block. Determining the source of this discordance is inherently complex. For example, the *B* allele of *Gabra1* has high expression in most array data sets, a finding that we cannot validate using either allele-specific expression assays or RNA-seq. In the case of *Gabrg2*, *Gabrb2*, and *Gabrb3*, alleles with higher expression are dependent on tissue, platform, and mRNA target region. A parsimonious explanation is the expression of a diverse repertoire of isoforms—a finding that is supported by the existence of alternative isoforms for many of the *cis* modulated subunits, including *Gabrb2* and *Gabrg2* [18], [19]. Hundreds of *B* vs. *D* polymorphisms in *Gabra1*, *Gabrb2*, *Gabrb3*, and *Gabrg2* may modulate isoform usage and expression among strains, highlighting the need for a complete molecular dissection to determine their impact.

### Distant modulation of GABA(A)R subunits and genetic independence

*Trans* eQTLs are responsible for a significant level of genetic variation in subunits, even when expression is also under the control of a strong *cis* eQTL (Figure 1 and Table S2). For example, many of the *cis*-regulated subunits—*Gabra1*, *Gabra2*, *Gabrb2*, and *Gabrb3*—are co-controlled by one or more *trans* eQTLs. In addition, subunits without strong *cis*-modulation such as *Gabrg1* and *Gabra5*, are strongly modulated by at least one *trans e*QTL.

Despite substantial coexpression among these subunits—especially for those comprising the major receptor subtype (*Gabra1*, *Gabrb2*, and *Gabrg2*)—individual subunits do not control the expression of other subunits either directly or indirectly (Figure 1). Due to the complex evolutionary history of vertebrate genomes, most GABA(A)R subunit genes are organized into clusters. In mouse, the major clusters reside on Chrs 5, 7, 11, and X. Each contains a single γ, one or two α, and a single β subunit. We expected evidence of receptor-to-receptor genetic co-modulation in the form of *trans* eQTLs with locations corresponding to the GABA(A) gene clusters. Remarkably, sequence variants and strong *cis* eQTLS within each cluster do not influence the expression of subunits in other clusters. While we do see strong *cis* eQTLs in some of these clusters there is no evidence of genetic “cross-talk” among clusters. All *trans* eQTLS were located in regions of the genome that do not contain any GABA(A)R genes (Figure 2). We also rarely observe a high level of coexpression (r>0.6) between GABA(A)R subunits and genes known to be involved in receptor trafficking, such as gephyrin, *GABARAP*, *Bdnf*, neurexin, neuroligin, protein kinase A and C, *Trak1*, and others (Table S9). Sequence variants in these genes do not contribute to variation in subunit expression. Our findings demonstrate that there is a small set of novel and still undefined genes that modulate GABA(A)R expression.

### Novel candidate QTLs and gene variants modulate subunit expression

*Rfk*, *Son*, and *Kbtbd11* are the three best candidate genes modulating *Gabra5*, *Gabra1*, and *Gabrb2* expression in the BXD hippocampus, respectively. *Rfk* is a TNF-receptor-1 and p22 (phox) binding protein that mediates NADPH oxidase activation and reactive oxygen species signaling [20]. *Son* is thought to be involved in mRNA processing [21], [22], [23]. Although, highly correlated with the expression of *Gabrb2* probe sets in both mouse and humans, the function of *Kbtbd11* is unknown. *Gabrg1* has three good candidate genes—*Atp8a2*, *Ebpl*, and *Tnfrsf19*. *Atp8a2* and *Tnfrsf19* play a role in lipid transport across membranes and signaling pathways associated with myelination and axon regeneration, respectively [24], [25]. Mutations in *ATP8A2* have been associated with mental retardation [26]. The functions of *Ebpl* are unknown. Finally, *Gabra2* is modulated by a *trans* eQTL on chromosome 10 at ∼86 Mb that aligns precisely with a QTL that controls variation in reversal learning among BXD strains [27]. Laughlin and colleagues nominated *Hcfc2*, *Syn3* and *Nt5dc3* as strong candidates but *Hcfc2*, a gene with unknown functions, has the highest correlation with α2 expression in mouse and human.

### Master QTLs that modulate expression of multiple subunits

Several loci are *trans* eQTLs for multiple subunits. In mouse hippocampus (database accession number GN110) three unlinked loci on Chr 5 modulate expression of nine subunits including *Gabra1*, *Gabra2*, *Gabra5*, *Gabra3*, *Gabrb1*, *Gabrb2*, *Gabrb3*, *Gabrg1*, and *Gabrg2* (Figure S4). The most proximal QTL modulates *Gabra5* and the last coding exon and proximal to middle 3′ UTR of *Gabrb3*. *Naaa* and *Cox18*, genes involved in endocannabinoid metabolism [28], [29] and cytochrome c oxidase assembly [30] respectively, are excellent candidates. *COX18* (227442_at) expression is correlated with expression of the middle 3′ UTR of *GABRA5* (206456_at, *r* = 0.41) and the distal 3′ UTR of *GABRB3* (227690_at, *r* = 0.42). Although COX18 is thought to be involved in assembly of mitochondrial respiratory chain complexes, it may also be involved in other undiscovered biological processes. In normal human brain (database accession number GN314), NAAA (215178_x_at) expression is highly correlated with both GABRA5 (215531_s_at, −0.62) and GABRB3 (227690_at, −0.62). NAAA, along with FAAH, is involved in the degradation of N-acylethanolamines—endogenous endocannabinoids that include the cannabinoid receptor type 1 (CB1) agonist anandamide [31]. Many GABAergic neurons and synapses express CB1 receptors [32], [33]. Endocannabinoids can modulate GABAergic neurotransmission, through both CB1 receptor-dependent [34], [35] and -independent mechanisms [36]. FAAH is generally considered to be the major enzyme responsible for endocannabinoid degradation—especially anandamide—in brain [37] but our data suggest a novel role for NAAA at GABAergic synapses.

The middle QTL on Chr 5 modulates expression of the middle to distal parts of the 3′ UTRs of four subunits—*Gabra1*, *Gabrb1*, *Gabrb2*, and *Gabrg2*. Numerous complex traits map to this part of Chr 5, which has been identified previously as a QTL for adult hippocampal neurogenesis and morphology of the infrapyramidal mossy fiber tract [38]. Three strong candidates in this region are involved in cell signaling (*Nos1* and *Rasal1*) [39] and transcriptional regulation (*Thrap2* or *Med13l*) [40], [41]. Krebs and colleagues also nominated *Nos1* based on previous associations between *Nos1* expression and neurogenesis and morphological variation. In human brain (database accession number GN314), multiple *NOS1* probe sets targeting different mRNA regions (239132_at, 207309_at, 207310_s_at, and 1560974_s_at) exhibit negative and positive correlations (|r|>0.4) with the expression of multiple *GABRA1* (244118_at), *GABRB1* (207010_at), *GABRB2* (1557122_s_at and 207352_s_at), and *GABRG2* (1568612_at) probe sets targeting mainly the middle to distal 3′ UTR. Much research has linked nitric oxide synthase activity with GABA levels and GABAergic neuronal subpopulations in hippocampus and other regions [42], [43], [44], [45]. However, the same positive or negative correlation structure (|*r*|>0.46) is observed for *MED13L* probe sets (212207_at, 212209_at, 242911_at) with the exception of the *GABRB1* probe set which is less well correlated (*r* = −0.35). In addition, *MED13L* is a RNA polymerase II transcriptional coactivator and could be involved in the regulation of subunit expression. In contrast, the expression of *RASAL1* (219752_at) is strongly and negatively correlated (*r*>−0.5) with *GABRB2* (242344_at and 1557122_s_at) only.

he distal QTL on Chr 5 identified by Overall and colleagues is a major *trans* regulatory locus in the hippocampus *(Trans5a)* that controls the expression of multiple GABA(A)R subunits and hundreds of other transcripts. Both our study and that of Overall and colleagues highlights *Zkscan1* as an excellent candidate gene. Multiple probe sets target different regions of *Zkscan1* mRNA. Probe set 1447944_at measures expression from the proximal 3′ UTR and is controlled by a strong *cis* eQTL. This probe set varies significantly among BXD strains (2.45-fold) and is highly correlated (*r*>0.6) with the expression of multiple GABA(A)R subunits including, *Gabra1*, *Gabrb2*, *Gabrb3*, and *Gabrg2*. Several insertions and deletions within *Zkscan1* and just upstream of the 5′ UTR could account for the enormous variation in expression among strains. In human brain (database accession number GN314), *ZKSCAN1* (214670_at and 1557953_at) is highly correlated (*r*>|0.5|) with *GABRA1* (244118_at), *GABRA2* (207014_at and 1554308_s_at), *GABRG1* (1552943_at), and *GABRG2* (1568612_at) and modestly correlated (|*r*|>0.35) with *GABRB2* (207352_s_at) and *GABRB3* (227690_at and 229724_at). Almost nothing is known about the function of *Zkscan1* but our data implicate this gene as a high priority target for functional validation.

In summary, we have identified strong candidate genes that modify the expression of one or more GABA(A)R subunits. We often observe differential genetic control corresponding to different mRNA regions of the same subunit. In general, correlations between candidate genes and GABA(A)R subunits are preserved across species. Identifying the causal gene in *trans* regulatory regions is likely to be more important for understanding GABA(A)R subunit regulation than identifying specific causal variants underlying a *cis* eQTL. This is because molecular networks will be much better conserved between species than specific sequence polymorphisms.

### Prevalence of 3′ UTR isoforms

The Affymetrix M430 array is unusual in that the designer, Dr. David Kulp, intentionally selected multiple probe sets to target both proximal and distal regions of the 3′ UTR. This design is ideal for comparative analysis of UTR variants that are associated with major differences in mRNA trafficking [46] and higher order function [8]. CNS transcripts have unusually long 3′ UTRs and tend to utilize alternative and more distal polyadenylation sites [47], [48]. For example, the 3′ UTR of *Gabrb2* is nearly 6 Kb long—4 times longer than the protein coding sequence. We also see multiple consensus sites for polyadenylation, and evidence of longer and shorter 3′ UTR isoforms for *Gabra1*, *Gabrb2*, and *Gabrg2* in our whole brain RNA-seq data (Figure S5, S6, S7).

An intriguing trend emerges from our dissection of GABA(A)R subunit expression in which probe sets targeting similar mRNA regions of different subunits are well correlated (Figure 4 and Table S4). This is especially true for the major receptor subtype—*Gabra1*, *Gabrb2*, and *Gabrg2*. Poor or negative correlations between probe sets representing different mRNA regions of the same subunit and high positive correlations between probe sets from different subunits that target similar regions are replicated across brain tissues from BXD strains as well as across human brain samples (database accession number GN314) (Table S5 and Figure S2). We asked if these coexpression patterns were part of a more general transcriptional network in the hippocampus of BXD strains (database accession number GN110). Probe sets targeting more distal 3′ UTR regions generally have higher correlations with other probe sets targeting distal regions whereas probe sets targeting the last few coding exons and proximal 3′ UTR generally have higher correlations with other probe sets targeting these proximal regions (Table S9). This pattern of coexpression—short to short, and long to long—may be related to differences in mRNA processing based on 3′ UTR length. Variation at the 3′ end of mRNA can expose alternate miRNA and protein binding sites that change localization, stability, or translation efficiency (reviewed in [49]). Another intriguing possibility that could explain the discordant expression between 3′ UTR and coding regions is the expression of distinct RNA transcripts from within the 3′ UTR [50]. Mercer and colleagues mined RIKEN CAGE data to identify genes producing full-length transcripts from the 3′ UTR, including *Gabra1*, *Gabra6*, *Gabra3*, and *Gabrb2* (mouse) and *GABRA2*, *GABRB1*, and *GABRB2* (human). Within this gene category and in 54% of the cases, expression of the 3′ UTR and coding exon is discordant. Further molecular analysis of 3′ UTR expression and new high-throughput methods for quantifying the 3′ end of mRNA will be needed to resolve which mechanisms contribute to the divergent expression patterns we observe among GABA(A)R subunit mRNA regions. How these isoforms contribute to cell and synapse type, circuitry, and disease will be a critical aspect of future studies.

### Functional impact of subunit variation and relevance to human disease

There is a lack of association between *cis* modulated GABA(A)R subunits and classic phenotypes generally linked with GABAergic signaling, including alcohol-related, anxiety, and pharmacological measures. Of course not every trait associated with these receptors has been measured among BXD strains and several physiological phenotypes, including receptor density and neuronal excitability are notable absent. However, the lack of association with available traits is still surprising given that as little as a 6 to 35% reduction in receptor density can produce robust phenotypic differences [51], [52]. The sole exception is for β3, a subunit that is genetically linked to mouse behaviors associated with anxiety. Deletion of *Gabrb3* leads to an increase in stretch-attend posturing—an phenotype associated with risk assessment [53]. Deletions of the 15q11-13 region in humans—including *GABRB3*—are associated with Angelman syndrome and severe cognitive deficits, a constellation of phenotypes matched in *Gabrb3*-deficient mice [54], [55].

The variation level of many GABA(A)R subunits is very high among individual strains and yet the functional impact on classic phenotypes—behavior and neuropharmacological traits, in particular—is minimal. In a normal human population we observe even greater differences in expression of these subunits—up to 100-fold in adults. Individual humans are highly polymorphic and a small portion of this variation could result from platform-dependent artifacts caused by hidden variants. However, whole genome and transcriptome sequencing technologies are rapidly advancing and our findings suggest that a high level of expression variation will persist even after these types of technical errors are corrected. This is an important negative finding that demonstrates the impressive ability of higher order network properties to buffer the variation of their constitutes. Much of this buffering may occur during translational processing and a 10-fold difference in transcript may be muted at the protein level. How molecular networks buffer, exploit, or accommodate high levels of variation in transcript expression is a major question that has not yet been addressed.

Why study variation in expression that does not cause prominent differences in phenotype? It is rare to find a natural variant that produces a null or fully penetrant phenotype. The subtle push and pull of modest gene variants on expression is a natural perturbation that exposes the large-scale structure of networks of genes and molecules. Understanding network structure and relations is highly relevant for understanding complex traits and disease risk. We identified members of the GABA(A)R network that are conserved across rodents and humans (Figure 5). These members are involved in signal transduction (*Rgs4*, *Mapk9*, *Socs5*) and synaptic transmission (*Syt1*) and are likely to be important for the function of GABAergic synapses. In the hippocampus, this network is under genetic control and expression is strongly modulated by the *Trans5a* QTL.

We also examined the set of conserved expression correlates of five or more GABA(A)R subunits and identified genes with the highest correlation levels in both human and mouse brain. These genes are likely to be important for subunit expression at GABAergic synapses and include, *Lis1*, *Gucy1a3* and *Gucy1b3* (Table S7, Table S8). *Lis1* is strongly associated with expression of many subunits in both species and is a member of the conserved GABA(A)R network. During development *Lis1*—along with GABA and other signaling molecules—is a key mediator of neuronal migration [56], [57]. Knockout mice have cortical and hippocampal defects and heterozygotes exhibit alterations in CA1 inhibitory circuits implicating dysfunction of the GABAergic system [58], [59]. GUCY1A3 (α1) and GUCY1B3 (β1) are subunits of soluble guanylyl cyclase, the main receptor for the retrograde signaling molecule nitric oxide (NO). NO can enhance GABAergic signaling through activation of presynaptic guanylyl cyclase and production of cGMP [60], [61], [62], [63].

### Conclusion

Our comprehensive analysis highlights the way in which expression variation and covariation can be used to identify strong candidates and novel biological processes that play a critical role in GABA(A)R mediated inhibitory transmission. We now have ample evidence of profound alternative 3′ UTR expression for many subunits in human and mouse brain. Elucidating the molecular regulation and functional consequences of these expression patterns is of the utmost importance. We also have identified a large and surprising list of regulatory genes and network members that are highly correlated with subunit expression in human and mouse brain. Most of these candidates are novel and suggest new and interesting modes of organization that influence GABA(A)R expression in brain.

## Materials and Methods

### Ethics Statement

All animal work was conducted according to an approved animal use protocol (UTHSC680) and in accordance with procedures approved by the Executive Committee of the Institutional Animal Care and Use Committee at The University of Tennessee Health Science Center.

### Database Descriptions

Detailed information and free access to all human and mouse data is provided at www.genenetwork.org. Mouse brain data sets include “Hippocampus Consortium M430v2 (Jun06) RMA” (accession number GN110). This data set incorporates expression profiles from 67 BXD RI strains, B6 and D2 parental strains and reciprocal F1 hybrid strains, and 15 inbred strains (mouse diversity panel). The “UMUTAffy Hippocampus Exon (Feb09) RMA” (accession number GN206) data set includes expression profiles from 72 BXD strains, B6 and D2 parental strains, and reciprocal F1 hybrid strains. The “Hippocampus Illumina (May07) RankInv” (accession number GN133) data set includes expression profiles from parental strains, ILS/Ibg (Inbred Long Sleep) and ISS/Ibg (Inbred Short Sleep), and 75 of their inbred progeny (LXS strains), and reciprocal F1 strains. The “OX UK HS ILM6v1.1 Hippocampus (May 2010) RankInv” (accession number GN268) data set includes expression profiles from 465 heterogeneous stock mice [64]. Data from other crosses include brain expression data measured on the Agilent platform among an F2 intercross between CAST/EiJ and C57BL/6J (accession number GN171) and among an F2 intercross between C57BL/6J and C3H/HeJ (accession number GN166). Other data sets include expression profiling on various microarray platforms among the BXD family of strains in the cerebellum (accession number GN56), whole brain (accession number GN123) [65], striatum (accession number GN298), neocortex (accession number GN282), amygdala (accession number GN280), and hypothalamus (accession number GN281).

Briefly, human brain tissue data sets include, “GSE5281 Human Brain Normal Full Liang (Jul09) RMA” (accession number GN314), “GSE15222 Human Brain Normal Meyers (Apr09) RankInv” (accession number GN234), and Harvard Brain Tissue Resource Center “HBTRC-MLC Human Cerebellum Agilent Normal (Jun11) mlratio” (accession number GN361). For data set GN314, cases include 16 normal aged subjects [10], [66], and gene expression was profiled for six regions on the Affymetrix platform. For data set GN234, expression was profiled for temporal cortex and cortical tissue from 187 normal aged adults on the Illumina Sentrix Bead array (HumanRef-8) using Illumina's rank invariant transform [67]. Data set GN361 includes expression profiles for cerebellum, visual cortex, and prefrontal cortex from 170 normal subjects using a custom-made Agilent 44 K microarray. Only expression in the cerebellum was used for comparison of rodent and human coexpression. This data set was contributed by Merck Pharmaceutical through the Sage Bionetworks Repository.

### Removal of probe sets overlapping B6 and D2 sequence variants

All 19 known GABA(A) subunit genes are represented by at least one probe set on the Affymetrix M430 2.0 and Exon 1.0 ST platforms. The former is designed to produce consensus estimates of expression across multiple mRNA isoforms using probes that target the 3′ UTR and the last few coding exons. In contrast, the exon array is designed to target every exon. Illumina probes generally target a single region from the last coding exons or the 3′ UTR. Probe target sequences corresponding to GABA(A) probe sets for Illumina and exon hippocampus BXD data sets were matched to all B6 and D2 SNPs and small indels. The offending probe set was removed every time an overlap occurred. For the M430 data we filtered out probes overlapping polymorphisms using tools in GeneNetwork. We used the remaining probe values and principal component analysis to construct corrected probe set values from the first principal component (PC1). B6 vs. D2 sequence variants were generated at UTHSC by comparison of the B6 reference genome and the D2 genome (100× whole genome coverage). This level of coverage was achieved using data generated from both the SOLiD and Illumina sequencing platforms and exceeds that of any other publicly available variant database. Variant data can be visualized by gene at ucscbrowser.genenetwork.org or by probe set using the RNA-seq tool in GeneNetwork. A list of all affected and unaffected probe sets is available in Table S1.

### Heritability

We calculated the broad-sense heritability to estimate the influence of genetic factors on variation in GABA(A)R subunit expression for the Hippocampus M430 data set. Heritability is the genetic variance (variance among strain means) divided by the total variance (variance among all measurements).

### RNAseq Analysis

Total RNA was isolated from the whole brain of B6, D2, 31 BXD, and two reciprocal F1 hybrid strains using the QIAGEN RNeasy kit. RNA quality was assessed using the Agilent Bioanalyzer 2100. Ribosomal RNA (rRNA) was removed from total RNA using the RiboMinus Eukaryote kit (Invitrogen). The SOLiD Whole Transcriptome Analysis kit was used to generate cDNA libraries for each strain. Each library was subsequently sequenced on the ABI SOLiD platform at the University of Tennessee Health Science Center. Data was analyzed using Applied Biosystems whole transcriptome software tools (http://www.solidsoftwaretools.com/). Reads are mapped to the mouse reference genome (mm9, National Center for Biotechnology Information (NCBI) build 37) with a minimum score of 24. Technical replicates were merged for each strain to produce a single BAM file (data available in our mirror of Galaxy at GeneNetwork, galaxy.genenetwork.org/library). The Cufflinks tool [68] was used within the Galaxy framework [69], [70] to generate gene level expression data based on RefSeq gene models (build 37) using a maximum intron length of 300,000, minimum isoform fraction of 0.1, and pre-mRNA fraction of 0.15.

### Identification of Candidate Genes in Upstream Regulatory Regions

Genes physically located within *trans* regulatory regions were filtered based on *cis*-modulation, coexpression within the tissue of interest, and covariation with the target GABA(A) subunit.

Expression in the hippocampus was confirmed for each candidate using the Allen Brain Atlas resource. Any candidate gene probe sets overlapping SNPs that also showed significantly higher expression associated with the *B* Allele were removed (see Ciobanu and colleagues 2010 for details). Candidates not polymorphic between B6 and D2 were also removed. Overlapping expression of the control and target gene in the tissue of interest and the presence of polymorphisms in the control gene locus are required conditions of nearly all *cis*-*trans* interactions as both genes can act within the same biological process or pathway [71]. If a candidate gene did not demonstrate highly significant *cis*-regulation (LRS>15) or covariation (*r*>|0.3|) with the target gene it was excluded from further analysis. Correlational analysis has also proven to be a useful tool to narrow the list of candidate genes [4], [72].

In general, multiple candidates were identified within each *trans* regulatory region using the above approach. Linkage between genes in close proximity can make it difficult to further narrow the list of candidate genes. To facilitate candidate gene identification we used the partial correlation feature available in GeneNetwork to determine the most likely set of *cis*-modulated genes within upstream regulatory regions that control target GABA(A) subunit expression. A partial correlation is the degree of association between a primary variable and a target variable after removing the effect of one or more control variables [73]. Specific details on partial correlations have been described in detail elsewhere [74]. In this case, the correlation between GABA(A)R subunit probe set expression (primary variable) and the expression of candidate regulatory gene probe sets (target variables) was measured after controlling for genetic variation at the peak *trans* QTL marker. We reasoned that, in the absence of genetic variation, residual biological variation would retain the correlation between the expression of the GABA(A)R subunit and the candidate regulatory gene. Therefore the candidate regulatory gene with the lowest reduction in the correlation between itself and GABA(A)R subunit expression is a superior candidate. Finally, covariation of the candidate control gene and the target subunit were examined in normal aged human brain samples (database accession number GN314) [10]. Correlations between control and target genes that are preserved across species are more likely to represent real biological networks of high translational relevance and were used to further prioritize candidate genes.

### Exon Coexpression

The “UMUTAffy Hippocampus Exon (Feb09) RMA” (accession number GN206) data set was used to investigate coexpression between expressed exons and introns for individual GABA(A)R subunits. As described previously, probe sets overlapping SNPs were removed and only those probe sets with an average log2 expression of 10 or greater were included in the analysis. Pair-wise Pearson and Spearman correlation coefficients were calculated among probe sets for each subunit using tools available in GeneNetwork. The correlation structure between probe sets was used as an indirect method to identify possible instances of alternative splicing—observed as a lack of correlation between probe sets targeting different exons of the same subunit. Probe sets from this array do not target exon-exon junctions and do not provide a direct measure of splicing.

### Cross-Species Coexpression

Modules (usually identified by a color) containing GABA(A) R subunits were identified from a weighted gene coexpression analysis of human and mouse data sets [16]. Most GABA(A) subunits had the highest correlation with the black and red module eigengenes in human and the tan module eigengene in mouse. Correlation with these three module eigengenes (R>0.3) was used to define coexpressed genes, including the GABA(A) subunits (GABRA1, GABRB2, GABRG2, and GABRB3). Overlap between the human and mouse module membership was determined by gene symbol. The expression of this consensus module of 28 genes was explored using the Hippocampus M430 database. A single probe set was selected for each gene based on correlation within the module. The first principal component captured ∼55% of the expression variation of module members and was used for QTL mapping. Genes from the human and mouse modules (*r*>0.3) were also compared to the results of a limited GeneNetwork cross-species survey to assess the amount of connectivity with other GABA(A) R subunits.

The criterion for consistent cross-species association was defined as: *r*≥|0.3| for three human (GN314, GN234, GN361) and three mouse brain data sets (GN110, GN133, GN268). Because of the number of samples within each database, this correlation is often highly significant (p<0.001).

## Supporting Information

Figure S1 **Pairwise comparison of platform expression for GABA(A)R subunits.** Scatter plots were constructed for the expression data from each platform shown in Table 1. All probe sets were used for this analysis. Pairwise correlations based on Pearson's *r* are shown at the top left-hand corner of each scatter plot. M430 (accession number GN110), exon (accession number GN206), and *in situ* data (Allen Brain Atlas Resource) are from the hippocampus while RNA-seq data is from whole brain. There is good agreement for the expression of most subunits as assayed across platforms. More modest correlations (∼0.5) are generally associated with comparisons between different tissue types—whole brain and hippocampus.
(TIF)

Figure S2 **Summary of GABA(A)R subunit coexpression in human brain.** Pairwise correlations based on Pearson's *r* are shown for each probe set combination in normal adult human brain (data available in GeneNetwork, accession number GN314). Boxes indicate low correlations between probe sets representing different regions of the same subunit. Positive and negative correlations are indicated by intensity of yellow and red shading, respectively.
(TIF)

Figure S3 **GABA(A)R subunit gene coexpression based on whole brain RNA-seq.** Bold lines indicate a correlation between 1 and 0.7 while normal and dashed lines indicate a correlation between 0.7 and 0.5 and 0.5 and 0.3, respectively. Warm colors represent positive correlations while cool colors represent negative correlations.
(TIF)

Figure S4 **Summary of genetic regulation of GABA(A)R subunit expression from Chr 5.** Probes that do not overlap a SNP or small insertion or deletion represent columns and ascending genomic location for Chr 5 represent rows. The position of the middle and distal (*Trans5a*) regulatory loci on Chr 5 are indicated by arrowheads to the left of each heat map. Hue intensity provides an indication of the strength of the association between gene expression and genomic location. Red and blue alleles indicate that the *D* or *B* allele increases trait values, respectively. Arrowheads show the location of each cognate gene. Disparate regulation of different mRNA regions associated with middle or distal Chr 5 loci is especially evident for *Gabra1*, *Gabrb2*, and *Gabrg2*. PC1 = first principal component, Chr = Chromosome, Mb = Megabase, Prox = proximal, mid = middle, dist = distal.
(TIF)

Figure S5 **Summary of** ***Gabra1*** **3′ UTR expression as assayed using RNA-seq.** Data available at ucscbrowser.genenetwork.org. *Gabra1* is located on the (−) strand. Tapering of reads from the proximal to more distal 3′ UTR indicates expression of longer and shorter 3′ UTR isoforms. This is especially prevalent in the striatum, which included only polyadenylated transcripts for RNA-seq. Track 1 shows the chromosomal location at top followed by gene model based on RefSeq as track 2. Larger width indicates coding exons and smaller width indicates 3′ UTR. All known variants between the B6 and D2 genome are indicated as part of track 3. Tracks 4 through 7 summarize the normalized (+) and (−) strand reads for the B6 (blue) and D2 (red) parental strains in whole brain (RiboMinus method). Tracks 8 and 9 show normalized reads in the striatum of 10 B6 and 11 D2 mice (PolyA enrichment method, [75]). For tracks 4 through 9 the scale to the left shows the read number, which will be lower for whole brain since it represents only a single RNA-seq run from one animal. Track 10 shows previously characterized mRNA species in mouse. Track 11 shows the degree of sequence conservation in mammals and Track 12 shows the presence of any repetitive DNA which is masked by most RNA-seq alignment algorithms.
(TIF)

Figure S6 **Summary of** ***Gabrb2*** **3′ UTR expression as assayed using RNA-seq.** Data available at ucscbrowser.genenetwork.org. *Gabrb2* is located on the (+) strand. Unequal distribution of reads in the 3′ UTR, especially evident in the striatum, could indicate alternative polyadenylation or splicing. Track 1 shows the chromosomal location at top followed by gene model based on RefSeq as track 2. Larger width indicates coding exons and smaller width indicates 3′ UTR. All known variants between the B6 and D2 genome are indicated as part of track 3. Tracks 4 through 7 summarize the normalized (+) and (−) strand reads for the B6 (blue) and D2 (red) parental strains in whole brain (RiboMinus method). Tracks 8 and 9 show normalized reads in the striatum of 10 B6 and 11 D2 mice (PolyA enrichment method, [75]). For tracks 4 through 9 the scale to the left shows the read number, which will be lower for whole brain since it represents only a single RNA-seq run from one animal. Track 10 shows previously characterized mRNA species in mouse. Track 11 shows the degree of sequence conservation in mammals and Track 12 shows the presence of any repetitive DNA which is masked by most RNA-seq alignment algorithms.
(TIF)

Figure S7 **Summary of** ***Gabrg2*** **3′ UTR expression as assayed using RNA-seq.** Data available at ucscbrowser.genenetwork.org. *Gabrg2* is located on the (−) strand. Tapering of reads from the proximal to more distal 3′ UTR indicates expression of longer and shorter 3′ UTR isoforms. This is especially prevalent in the striatum, which included only polyadenylated transcripts for RNA-seq. Track 1 shows the chromosomal location at top followed by gene model based on RefSeq as track 2. Larger width indicates coding exons and smaller width indicates 3′ UTR. All known variants between the B6 and D2 genome are indicated as part of track 3. Tracks 4 through 7 summarize the normalized (+) and (−) strand reads for the B6 (blue) and D2 (red) parental strains in whole brain (RiboMinus method). Tracks 8 and 9 show normalized reads in the striatum of 10 B6 and 11 D2 mice (PolyA enrichment method, [75]). For tracks 4 through 9 the scale to the left shows the read number, which will be lower for whole brain since it represents only a single RNA-seq run from one animal. Track 10 shows previously characterized mRNA species in mouse. Track 11 shows the degree of sequence conservation in mammals and Track 12 shows the presence of any repetitive DNA, which is masked by most RNA-seq alignment algorithms.
(TIF)

Table S1 Summary of GABA(A)R probe sets, expression, and overlapping variants in the hippocampus. Chr = Chromosome, Mb = Megabase, c = Coding, prox = proximal, dist = distal, Mean Expr = Mean Log2 Normalized Expression, FC = Fold Change, MRS = SNP, MINA = small insertion, Shading = Mean Expr>8 (M430) or >10 (Exon).
(XLSX)

Table S2 Summary of GABA(A)R heritability and eQTLs in the hippocampus. Expression (Expr), standard deviation (SD), and fold change(FC) calculated across all strains, including parents and F1. * = probes overlapping SNPs removed and probe set reconstructed as the first principal component (PC1). PC1 used to map expression quantatative trait loci (eQTL). Only eQTL that have achieved genome wide significance (∼LRS>15) are shown.
(XLSX)

Table S3 Summary of GABA(A)R exon coexpression in the hippocampus. Expression must be greater than 10 for all probe sets and probe sets overlapping SNPs were excluded from the analysis.
(XLSX)

Table S4 Probe set correlations in the hippocampus.
(XLSX)

Table S5 Probe set correlations in the whole brain and cerebellum.
(XLSX)

Table S6 Phenotypes mapping to middle and distal Chr 5 QTLs.
(XLSX)

Table S7 Summary of across species correlations between GABA(A)R subunits and Gucy1a3 and Gucy1b3.
(XLSX)

Table S8 Summary of across species correlations between GABA(A)R subunits and Pafah1b1/Lis1.
(XLSX)

Table S9 Summary of Gabra1, Gabrb2, and Gabrg2 exon and distal 3′ UTR probe set correlates in the hippocampus.
(XLSX)

## References

[1] Webster JA, Gibbs JR, Clarke J, Ray M, Zhang W (2009). Genetic control of human brain transcript expression in Alzheimer disease.. American journal of human genetics.

[2] Thompson CL, Pathak SD, Jeromin A, Ng LL, MacPherson CR (2008). Genomic anatomy of the hippocampus.. Neuron.

[3] Benovoy D, Kwan T, Majewski J (2008). Effect of polymorphisms within probe-target sequences on olignonucleotide microarray experiments.. Nucleic Acids Research.

[4] Ciobanu DC, Lu L, Mozhui K, Wang X, Jagalur M (2010). Detection, validation, and downstream analysis of allelic variation in gene expression.. Genetics.

[5] Walter NA, McWeeney SK, Peters ST, Belknap JK, Hitzemann R (2007). SNPs matter: impact on detection of differential expression.. Nat Methods.

[6] Damerval C, Maurice A, Josse JM, de Vienne D (1994). Quantitative trait loci underlying gene product variation: a novel perspective for analyzing regulation of genome expression.. Genetics.

[7] Korostynski M, Kaminska-Chowaniec D, Piechota M, Przewlocki R (2006). Gene expression profiling in the striatum of inbred mouse strains with distinct opioid-related phenotypes.. BMC Genomics.

[8] Li Z, Mulligan MK, Wang X, Miles MF, Lu L (2010). A transposon in comt generates mRNA variants and causes widespread expression and behavioral differences among mice.. PLoS One.

[9] Philip VM, Duvvuru S, Gomero B, Ansah TA, Blaha CD (2010). High-throughput behavioral phenotyping in the expanded panel of BXD recombinant inbred strains.. Genes Brain Behav.

[10] Liang X, Schnetz-Boutaud N, Bartlett J, Allen MJ, Gwirtsman H (2008). No association between SNP rs498055 on chromosome 10 and late-onset Alzheimer disease in multiple datasets.. Ann Hum Genet.

[11] Olsen RW, Sieghart W (2008). International Union of Pharmacology. LXX. Subtypes of gamma-aminobutyric acid(A) receptors: classification on the basis of subunit composition, pharmacology, and function. Update.. Pharmacol Rev.

[12] Olsen RW, Sieghart W (2009). GABA A receptors: subtypes provide diversity of function and pharmacology.. Neuropharmacology.

[13] Overall RW, Kempermann G, Peirce J, Lu L, Goldowitz D (2009). Genetics of the hippocampal transcriptome in mouse: a systematic survey and online neurogenomics resource.. Front Neurosci.

[14] Friebe A, Mergia E, Dangel O, Lange A, Koesling D (2007). Fatal gastrointestinal obstruction and hypertension in mice lacking nitric oxide-sensitive guanylyl cyclase.. Proc Natl Acad Sci U S A.

[15] Kholmanskikh SS, Dobrin JS, Wynshaw-Boris A, Letourneau PC, Ross ME (2003). Disregulated RhoGTPases and actin cytoskeleton contribute to the migration defect in Lis1-deficient neurons.. J Neurosci.

[16] Miller JA, Horvath S, Geschwind DH (2010). Divergence of human and mouse brain transcriptome highlights Alzheimer disease pathways.. Proc Natl Acad Sci U S A.

[17] Zhang B, Kirov S, Snoddy J (2005). WebGestalt: an integrated system for exploring gene sets in various biological contexts.. Nucleic Acids Research.

[18] McKinley DD, Lennon DJ, Carter DB (1995). Cloning, sequence analysis and expression of two forms of mRNA coding for the human beta 2 subunit of the GABAA receptor.. Brain research Molecular brain research.

[19] Whiting P, McKernan RM, Iversen LL (1990). Another mechanism for creating diversity in gamma-aminobutyrate type A receptors: RNA splicing directs expression of two forms of gamma 2 phosphorylation site.. Proceedings of the National Academy of Sciences of the United States of America.

[20] Yazdanpanah B, Wiegmann K, Tchikov V, Krut O, Pongratz C (2009). Riboflavin kinase couples TNF receptor 1 to NADPH oxidase.. Nature.

[21] Huen MS, Sy SM, Leung KM, Ching YP, Tipoe GL (2010). SON is a spliceosome-associated factor required for mitotic progression.. Cell Cycle.

[22] Sharma A, Takata H, Shibahara K, Bubulya A, Bubulya PA (2010). Son is essential for nuclear speckle organization and cell cycle progression.. Mol Biol Cell.

[23] Wynn SL, Fisher RA, Pagel C, Price M, Liu QY (2000). Organization and conservation of the GART/SON/DONSON locus in mouse and human genomes.. Genomics.

[24] Coleman JA, Kwok MC, Molday RS (2009). Localization, purification, and functional reconstitution of the P4-ATPase Atp8a2, a phosphatidylserine flippase in photoreceptor disc membranes.. J Biol Chem.

[25] Park JB, Yiu G, Kaneko S, Wang J, Chang J (2005). A TNF receptor family member, TROY, is a coreceptor with Nogo receptor in mediating the inhibitory activity of myelin inhibitors.. Neuron.

[26] Cacciagli P, Haddad MR, Mignon-Ravix C, El-Waly B, Moncla A (2010). Disruption of the ATP8A2 gene in a patient with a t(10;13) de novo balanced translocation and a severe neurological phenotype.. Eur J Hum Genet.

[27] Laughlin RE, Grant TL, Williams RW, Jentsch JD (2011). Genetic dissection of behavioral flexibility: reversal learning in mice.. Biol Psychiatry.

[28] Ueda N, Yamanaka K, Yamamoto S (2001). Purification and characterization of an acid amidase selective for N-palmitoylethanolamine, a putative endogenous anti-inflammatory substance.. The Journal of biological chemistry.

[29] Ueda N, Tsuboi K, Uyama T (2010). N-acylethanolamine metabolism with special reference to N-acylethanolamine-hydrolyzing acid amidase (NAAA).. Progress in lipid research.

[30] Sacconi S, Trevisson E, Pistollato F, Baldoin MC, Rezzonico R (2005). hCOX18 and hCOX19: two human genes involved in cytochrome c oxidase assembly.. Biochem Biophys Res Commun.

[31] Tsuboi K, Takezaki N, Ueda N (2007). The N-acylethanolamine-hydrolyzing acid amidase (NAAA).. Chemistry & biodiversity.

[32] Tsou K, Brown S, Sanudo-Pena MC, Mackie K, Walker JM (1998). Immunohistochemical distribution of cannabinoid CB1 receptors in the rat central nervous system.. Neuroscience.

[33] Katona I, Sperlagh B, Sik A, Kafalvi A, Vizi ES (1999). Presynaptically located CB1 cannabinoid receptors regulate GABA release from axon terminals of specific hippocampal interneurons.. The Journal of neuroscience: the official journal of the Society for Neuroscience.

[34] Hajos N, Katona I, Naiem SS, MacKie K, Ledent C (2000). Cannabinoids inhibit hippocampal GABAergic transmission and network oscillations.. The European journal of neuroscience.

[35] Kim J, Alger BE (2010). Reduction in endocannabinoid tone is a homeostatic mechanism for specific inhibitory synapses.. Nature neuroscience.

[36] Hofmann ME, Bhatia C, Frazier CJ (2011). Cannabinoid receptor agonists potentiate action potential-independent release of GABA in the dentate gyrus through a CB1 receptor-independent mechanism.. The Journal of physiology.

[37] Clement AB, Hawkins EG, Lichtman AH, Cravatt BF (2003). Increased seizure susceptibility and proconvulsant activity of anandamide in mice lacking fatty acid amide hydrolase.. The Journal of neuroscience: the official journal of the Society for Neuroscience.

[38] Krebs J, Romer B, Overall RW, Fabel K, Babu H (2011). Adult Hippocampal Neurogenesis and Plasticity in the Infrapyramidal Bundle of the Mossy Fiber Projection: II. Genetic Covariation and Identification of Nos1 as Linking Candidate Gene.. Front Neurosci.

[39] Jin H, Wang X, Ying J, Wong AH, Cui Y (2007). Epigenetic silencing of a Ca(2+)-regulated Ras GTPase-activating protein RASAL defines a new mechanism of Ras activation in human cancers.. Proc Natl Acad Sci U S A.

[40] Musante L, Bartsch O, Ropers HH, Kalscheuer VM (2004). cDNA cloning and characterization of the human THRAP2 gene which maps to chromosome 12q24, and its mouse ortholog Thrap2.. Gene.

[41] Sato S, Tomomori-Sato C, Parmely TJ, Florens L, Zybailov B (2004). A set of consensus mammalian mediator subunits identified by multidimensional protein identification technology.. Mol Cell.

[42] Harvey BH, Oosthuizen F, Brand L, Wegener G, Stein DJ (2004). Stress-restress evokes sustained iNOS activity and altered GABA levels and NMDA receptors in rat hippocampus.. Psychopharmacology (Berl).

[43] Jinno S, Kosaka T (2004). Patterns of colocalization of neuronal nitric oxide synthase and somatostatin-like immunoreactivity in the mouse hippocampus: quantitative analysis with optical disector.. Neuroscience.

[44] Li DP, Chen SR, Pan HL (2002). Nitric oxide inhibits spinally projecting paraventricular neurons through potentiation of presynaptic GABA release.. J Neurophysiol.

[45] Seress L, Abraham H, Lin H, Totterdell S (2002). Nitric oxide-containing pyramidal neurons of the subiculum innervate the CA1 area.. Exp Brain Res.

[46] An JJ, Gharami K, Liao GY, Woo NH, Lau AG (2008). Distinct role of long 3′ UTR BDNF mRNA in spine morphology and synaptic plasticity in hippocampal neurons.. Cell.

[47] Wang ET, Sandberg R, Luo S, Khrebtukova I, Zhang L (2008). Alternative isoform regulation in human tissue transcriptomes.. Nature.

[48] Zhang H, Lee JY, Tian B (2005). Biased alternative polyadenylation in human tissues.. Genome biology.

[49] Di Giammartino DC, Nishida K, Manley JL (2011). Mechanisms and consequences of alternative polyadenylation.. Molecular cell.

[50] Mercer TR, Wilhelm D, Dinger ME, Solda G, Korbie DJ (2011). Expression of distinct RNAs from 3′ untranslated regions.. Nucleic Acids Research.

[51] Crestani F, Lorez M, Baer K, Essrich C, Benke D (1999). Decreased GABAA-receptor clustering results in enhanced anxiety and a bias for threat cues.. Nature neuroscience.

[52] Shen Q, Lal R, Luellen BA, Earnheart JC, Andrews AM (2010). gamma-Aminobutyric acid-type A receptor deficits cause hypothalamic-pituitary-adrenal axis hyperactivity and antidepressant drug sensitivity reminiscent of melancholic forms of depression.. Biological psychiatry.

[53] Hashemi E, Sahbaie P, Davies MF, Clark JD, DeLorey TM (2007). Gabrb3 gene deficient mice exhibit increased risk assessment behavior, hypotonia and expansion of the plexus of locus coeruleus dendrites.. Brain Res.

[54] DeLorey TM, Handforth A, Anagnostaras SG, Homanics GE, Minassian BA (1998). Mice lacking the beta3 subunit of the GABAA receptor have the epilepsy phenotype and many of the behavioral characteristics of Angelman syndrome.. J Neurosci.

[55] Williams CA, Angelman H, Clayton-Smith J, Driscoll DJ, Hendrickson JE (1995). Angelman syndrome: consensus for diagnostic criteria. Angelman Syndrome Foundation.. Am J Med Genet.

[56] Behar TN, Schaffner AE, Scott CA, Greene CL, Barker JL (2000). GABA receptor antagonists modulate postmitotic cell migration in slice cultures of embryonic rat cortex.. Cereb Cortex.

[57] LoTurco JJ, Bai J (2006). The multipolar stage and disruptions in neuronal migration.. Trends Neurosci.

[58] Jones DL, Baraban SC (2009). Inhibitory inputs to hippocampal interneurons are reorganized in Lis1 mutant mice.. J Neurophysiol.

[59] Jones DL, Baraban SC (2007). Characterization of inhibitory circuits in the malformed hippocampus of Lis1 mutant mice.. J Neurophysiol.

[60] Szabadits E, Cserep C, Szonyi A, Fukazawa Y, Shigemoto R (2011). NMDA receptors in hippocampal GABAergic synapses and their role in nitric oxide signaling.. The Journal of neuroscience: the official journal of the Society for Neuroscience.

[61] Nugent FS, Penick EC, Kauer JA (2007). Opioids block long-term potentiation of inhibitory synapses.. Nature.

[62] Stern JE, Ludwig M (2001). NO inhibits supraoptic oxytocin and vasopressin neurons via activation of GABAergic synaptic inputs.. American journal of physiology Regulatory, integrative and comparative physiology.

[63] Li DP, Chen SR, Pan HL (2002). Nitric oxide inhibits spinally projecting paraventricular neurons through potentiation of presynaptic GABA release.. Journal of neurophysiology.

[64] Huang GJ, Shifman S, Valdar W, Johannesson M, Yalcin B (2009). High resolution mapping of expression QTLs in heterogeneous stock mice in multiple tissues.. Genome Res.

[65] Saba L, Bhave SV, Grahame N, Bice P, Lapadat R (2006). Candidate genes and their regulatory elements: alcohol preference and tolerance.. Mammalian genome: official journal of the International Mammalian Genome Society.

[66] Liang X, Schnetz-Boutaud N, Kenealy SJ, Jiang L, Bartlett J (2006). Covariate analysis of late-onset Alzheimer disease refines the chromosome 12 locus.. Mol Psychiatry.

[67] Webster JA, Gibbs JR, Clarke J, Ray M, Zhang W (2009). Genetic control of human brain transcript expression in Alzheimer disease.. Am J Hum Genet.

[68] Trapnell C, Williams BA, Pertea G, Mortazavi A, Kwan G (2010). Transcript assembly and quantification by RNA-Seq reveals unannotated transcripts and isoform switching during cell differentiation.. Nat Biotechnol.

[69] Blankenberg D, Gordon A, Von Kuster G, Coraor N, Taylor J (2010). Manipulation of FASTQ data with Galaxy.. Bioinformatics.

[70] Blankenberg D, Von Kuster G, Coraor N, Ananda G, Lazarus R (2010). Galaxy: a web-based genome analysis tool for experimentalists.. Curr Protoc Mol Biol Chapter.

[71] Chesler EJ, Lu L, Shou S, Qu Y, Gu J (2005). Complex trait analysis of gene expression uncovers polygenic and pleiotropic networks that modulate nervous system function.. Nature genetics.

[72] Bing N, Hoeschele I (2005). Genetical genomics analysis of a yeast segregant population for transcription network inference.. Genetics.

[73] de la Fuente A, Bing N, Hoeschele I, Mendes P (2004). Discovery of meaningful associations in genomic data using partial correlation coefficients.. Bioinformatics.

[74] Mozhui K, Ciobanu DC, Schikorski T, Wang X, Lu L (2008). Dissection of a QTL hotspot on mouse distal chromosome 1 that modulates neurobehavioral phenotypes and gene expression.. PLoS Genet.

[75] Bottomly D, Walter NA, Hunter JE, Darakjian P, Kawane S (2011). Evaluating gene expression in C57BL/6J and DBA/2J mouse striatum using RNA-Seq and microarrays.. PLoS One.

