# Supplementary figures and images for: Complex Control of GABA(A) Receptor Subunit mRNA Expression: Variation, Covariation, and Genetic Regulation
Source: PLoS One. 2012 Apr 10;7(4):e34586. doi: 10.1371/journal.pone.0034586 (PMC3323555; doi:10.1371/journal.pone.0034586)

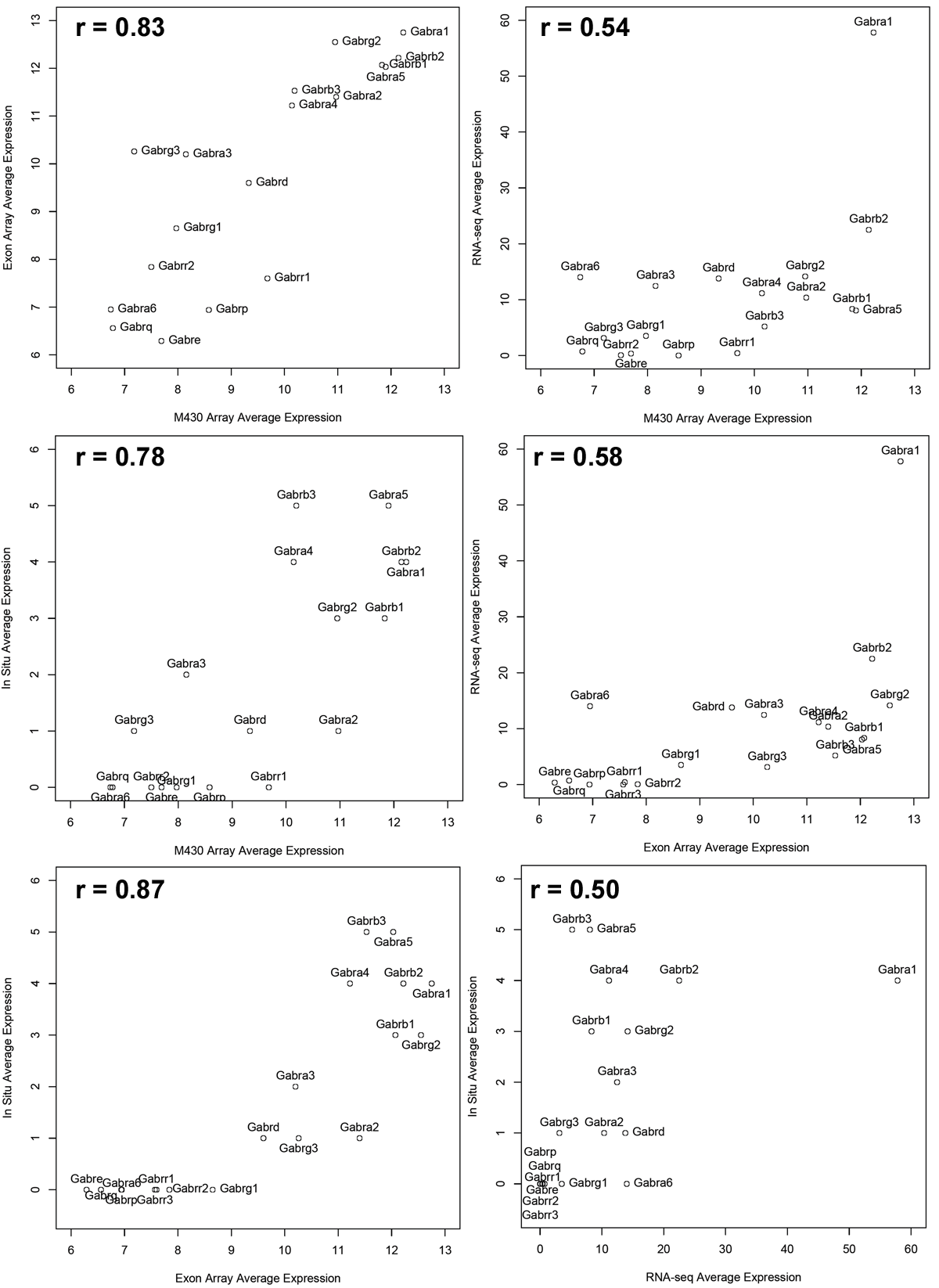

Supplement: Figure S1 — Pairwise comparison of platform expression for GABA(A)R subunits. Scatter plots were constructed for the expression data from each platform shown in Table 1. All probe sets were used for this analysis. Pairwise correlations based on Pearson's r are shown at the top left-hand corner of each scatter plot. M430 (accession number GN110), exon (accession number GN206), and in situ data (Allen Brain Atlas Resource) are from the hippocampus while RNA-seq data is from whole brain. There is good agreement for the expression of most subunits as assayed across platforms. More modest correlations (∼0.5) are generally associated with comparisons between different tissue types—whole brain and hippocampus. (TIF) [file pone.0034586.s001.tif]

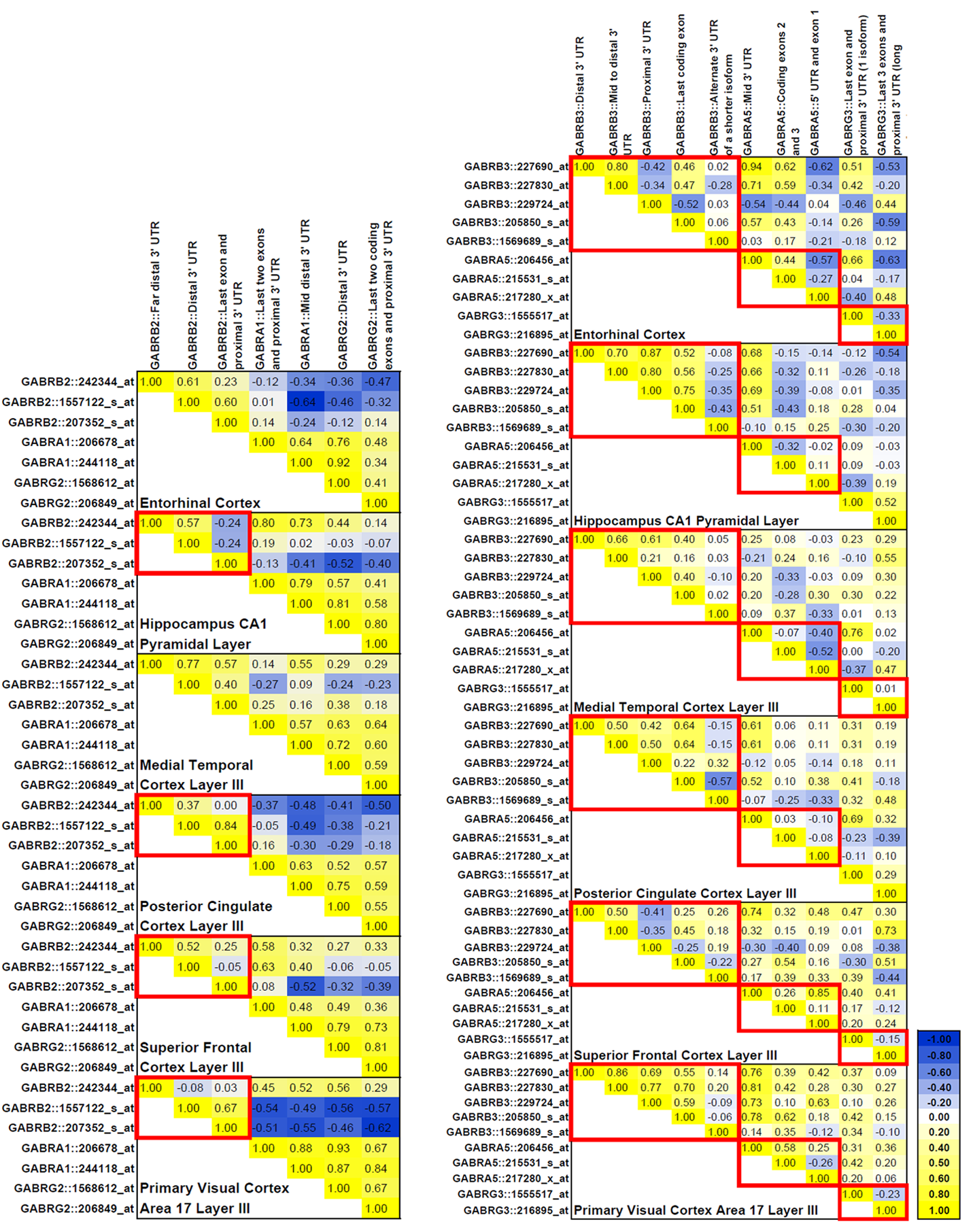

Supplement: Figure S2 — Summary of GABA(A)R subunit coexpression in human brain. Pairwise correlations based on Pearson's r are shown for each probe set combination in normal adult human brain (data available in GeneNetwork, accession number GN314). Boxes indicate low correlations between probe sets representing different regions of the same subunit. Positive and negative correlations are indicated by intensity of yellow and red shading, respectively. (TIF) [file pone.0034586.s002.tif]

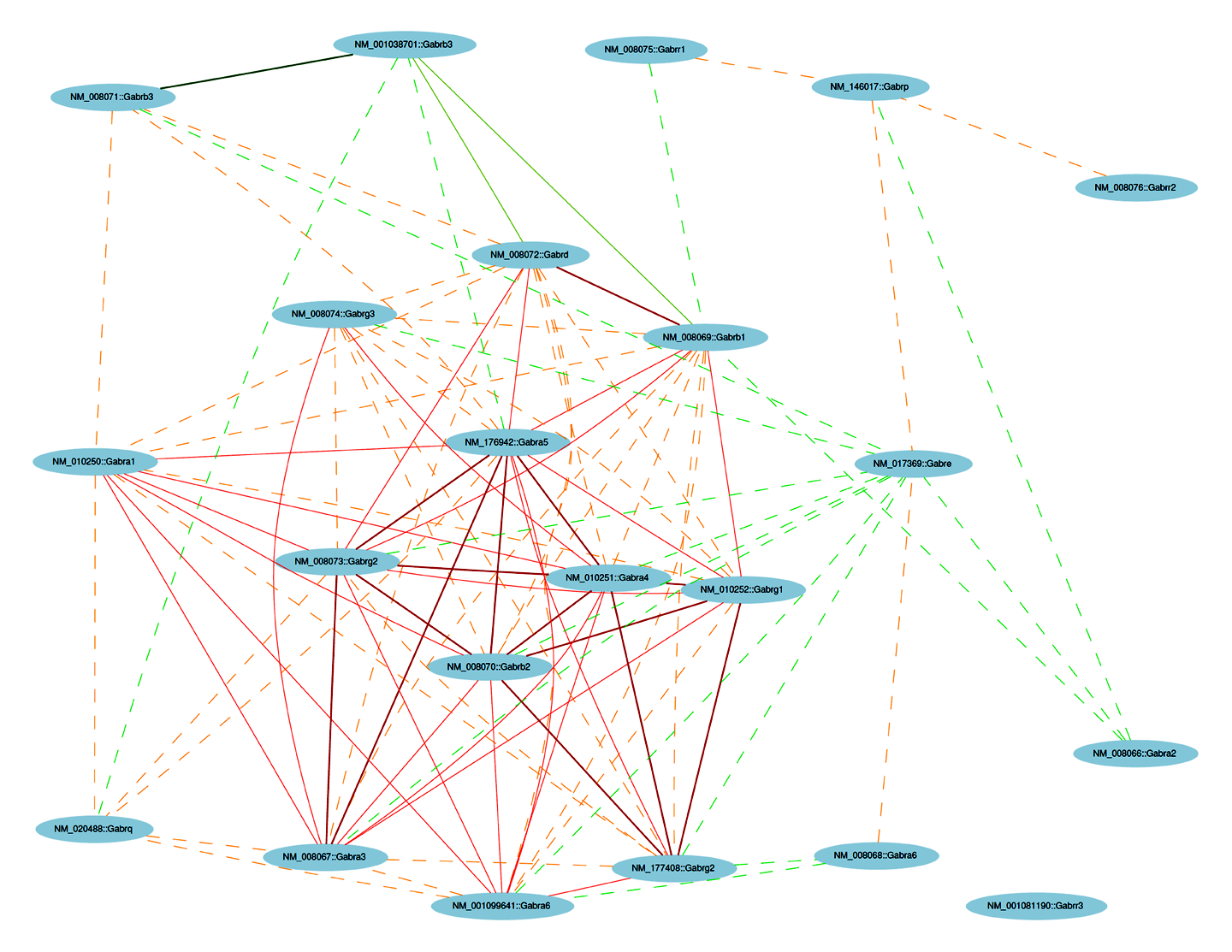

Supplement: Figure S3 — GABA(A)R subunit gene coexpression based on whole brain RNA-seq. Bold lines indicate a correlation between 1 and 0.7 while normal and dashed lines indicate a correlation between 0.7 and 0.5 and 0.5 and 0.3, respectively. Warm colors represent positive correlations while cool colors represent negative correlations. (TIF) [file pone.0034586.s003.tif]

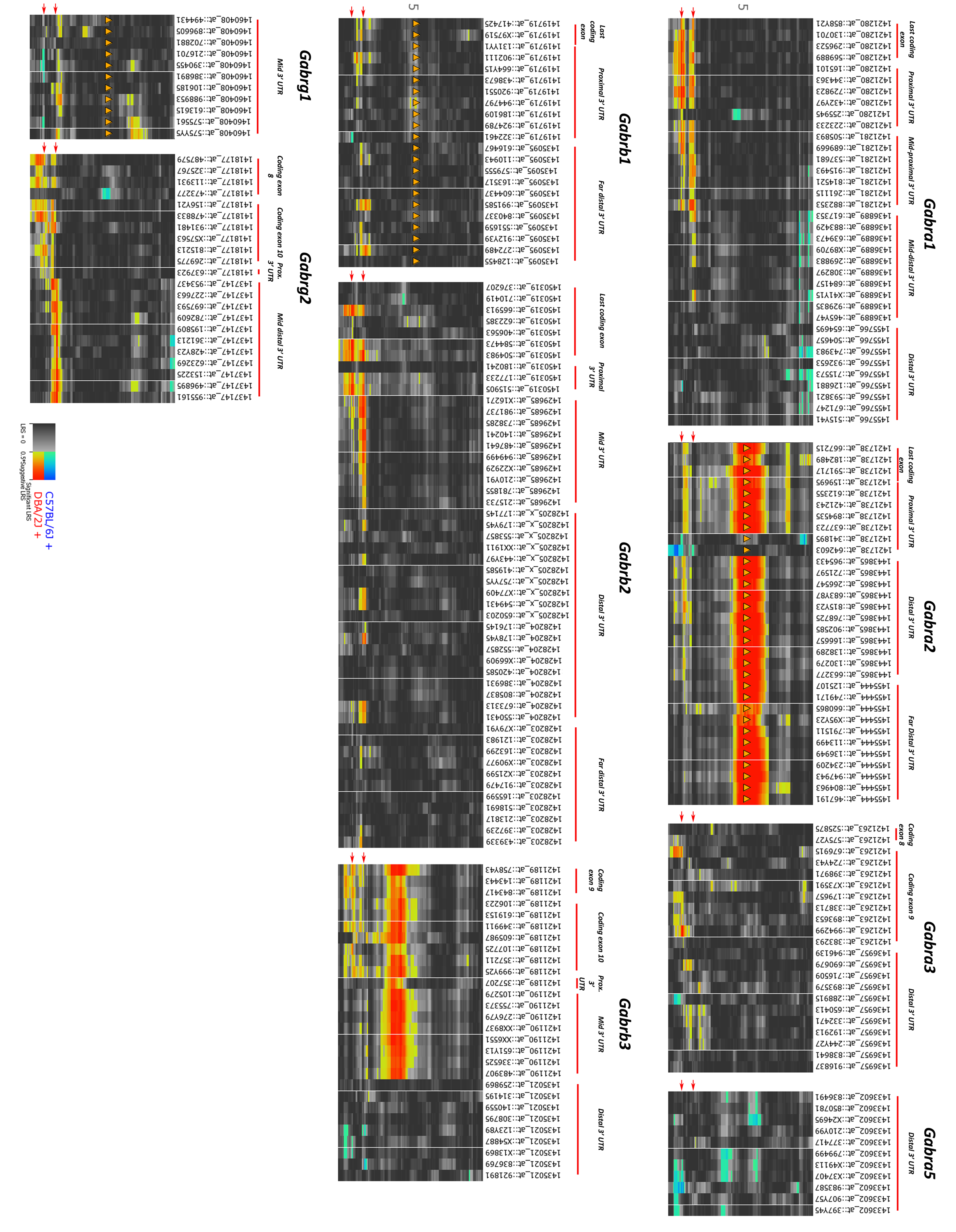

Supplement: Figure S4 — Summary of genetic regulation of GABA(A)R subunit expression from Chr 5. Probes that do not overlap a SNP or small insertion or deletion represent columns and ascending genomic location for Chr 5 represent rows. The position of the middle and distal (Trans5a) regulatory loci on Chr 5 are indicated by arrowheads to the left of each heat map. Hue intensity provides an indication of the strength of the association between gene expression and genomic location. Red and blue alleles indicate that the D or B allele increases trait values, respectively. Arrowheads show the location of each cognate gene. Disparate regulation of different mRNA regions associated with middle or distal Chr 5 loci is especially evident for Gabra1, Gabrb2, and Gabrg2. PC1 = first principal component, Chr = Chromosome, Mb = Megabase, Prox = proximal, mid = middle, dist = distal. (TIF) [file pone.0034586.s004.tif]

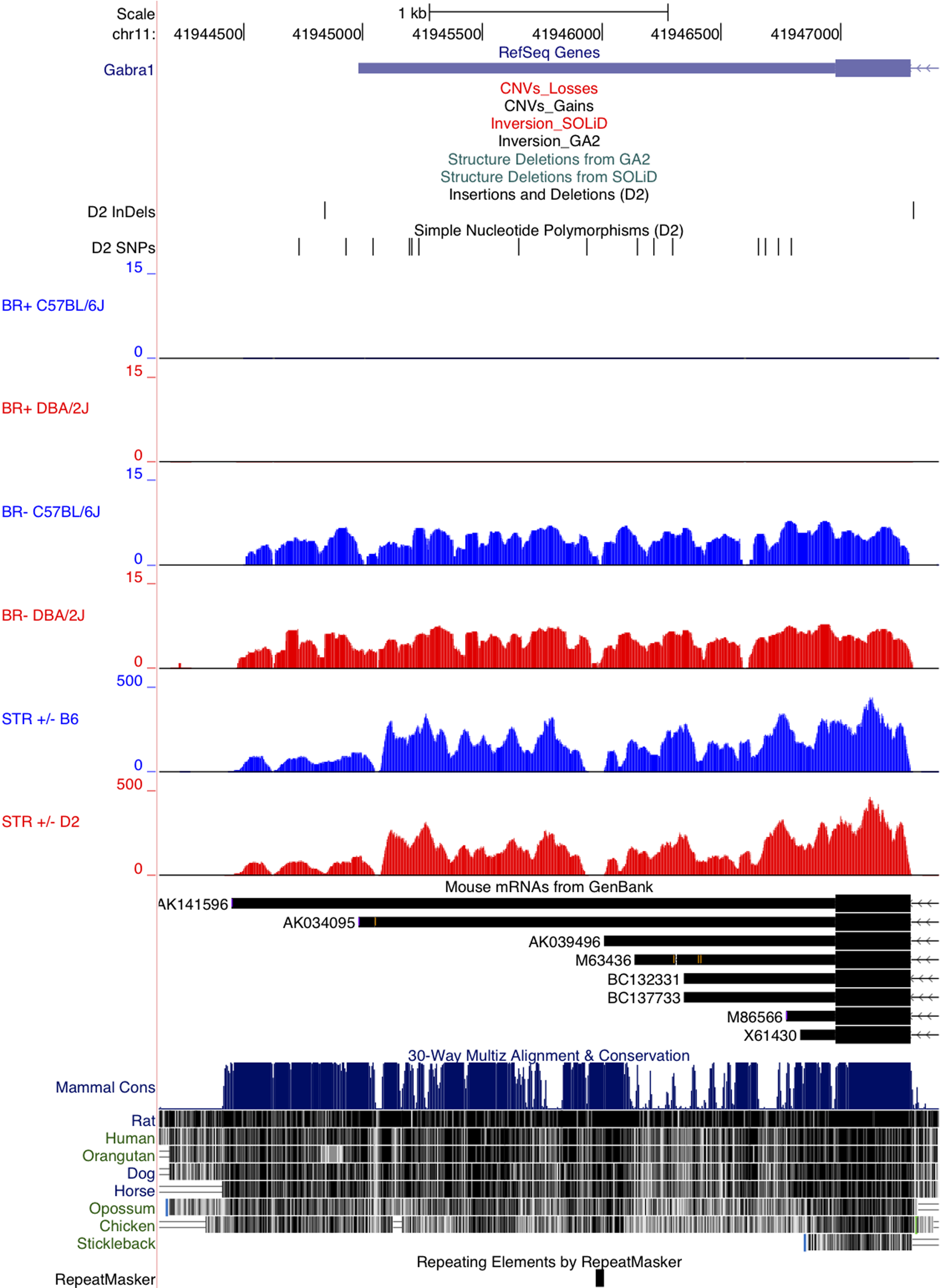

Supplement: Figure S5 — Summary of Gabra1 3′ UTR expression as assayed using RNA-seq. Data available at ucscbrowser.genenetwork.org. Gabra1 is located on the (−) strand. Tapering of reads from the proximal to more distal 3′ UTR indicates expression of longer and shorter 3′ UTR isoforms. This is especially prevalent in the striatum, which included only polyadenylated transcripts for RNA-seq. Track 1 shows the chromosomal location at top followed by gene model based on RefSeq as track 2. Larger width indicates coding exons and smaller width indicates 3′ UTR. All known variants between the B6 and D2 genome are indicated as part of track 3. Tracks 4 through 7 summarize the normalized (+) and (−) strand reads for the B6 (blue) and D2 (red) parental strains in whole brain (RiboMinus method). Tracks 8 and 9 show normalized reads in the striatum of 10 B6 and 11 D2 mice (PolyA enrichment method, [75]). For tracks 4 through 9 the scale to the left shows the read number, which will be lower for whole brain since it represents only a single RNA-seq run from one animal. Track 10 shows previously characterized mRNA species in mouse. Track 11 shows the degree of sequence conservation in mammals and Track 12 shows the presence of any repetitive DNA which is masked by most RNA-seq alignment algorithms. (TIF) [file pone.0034586.s005.tif]

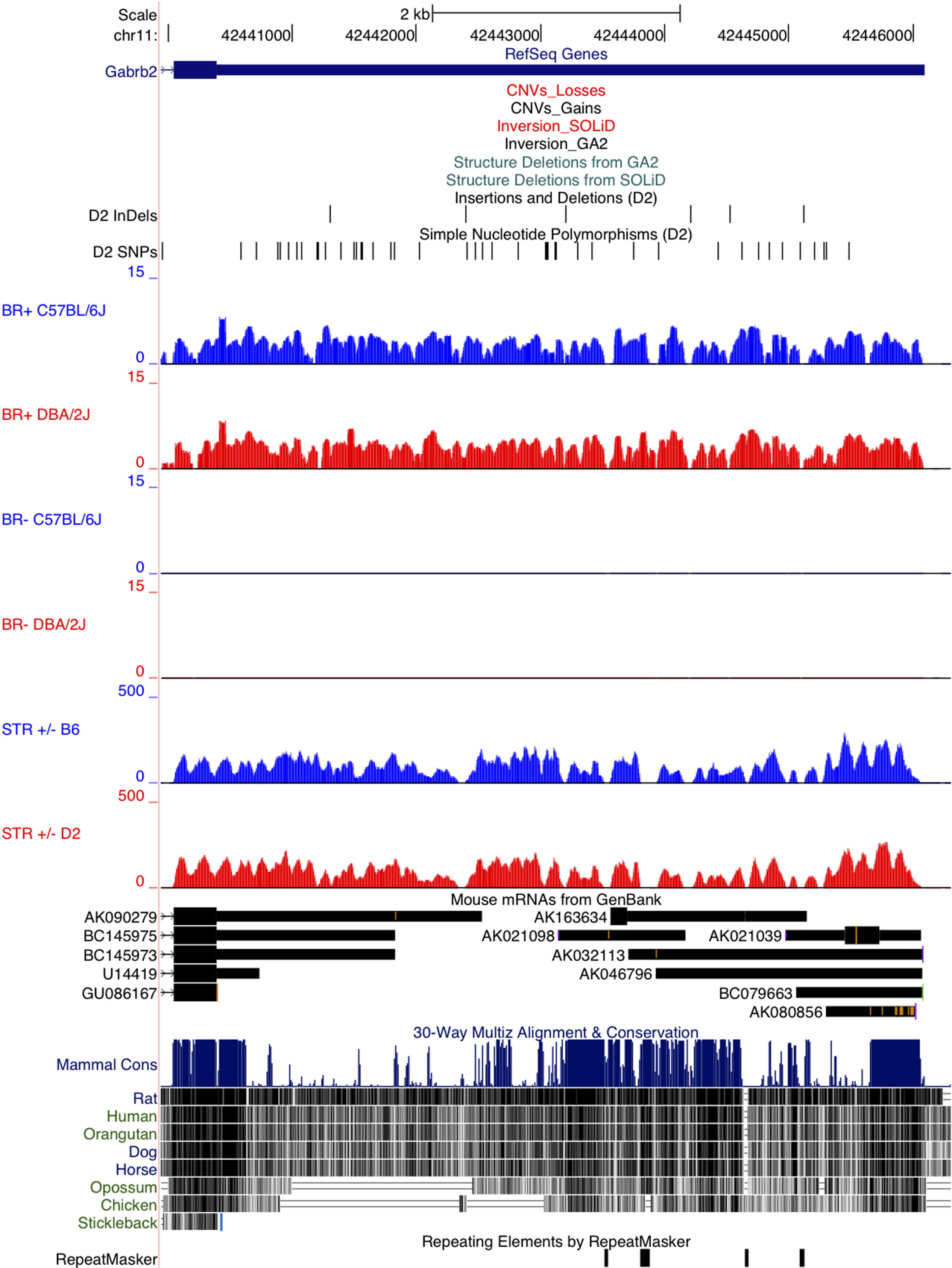

Supplement: Figure S6 — Summary of Gabrb2 3′ UTR expression as assayed using RNA-seq. Data available at ucscbrowser.genenetwork.org. Gabrb2 is located on the (+) strand. Unequal distribution of reads in the 3′ UTR, especially evident in the striatum, could indicate alternative polyadenylation or splicing. Track 1 shows the chromosomal location at top followed by gene model based on RefSeq as track 2. Larger width indicates coding exons and smaller width indicates 3′ UTR. All known variants between the B6 and D2 genome are indicated as part of track 3. Tracks 4 through 7 summarize the normalized (+) and (−) strand reads for the B6 (blue) and D2 (red) parental strains in whole brain (RiboMinus method). Tracks 8 and 9 show normalized reads in the striatum of 10 B6 and 11 D2 mice (PolyA enrichment method, [75]). For tracks 4 through 9 the scale to the left shows the read number, which will be lower for whole brain since it represents only a single RNA-seq run from one animal. Track 10 shows previously characterized mRNA species in mouse. Track 11 shows the degree of sequence conservation in mammals and Track 12 shows the presence of any repetitive DNA which is masked by most RNA-seq alignment algorithms. (TIF) [file pone.0034586.s006.tif]

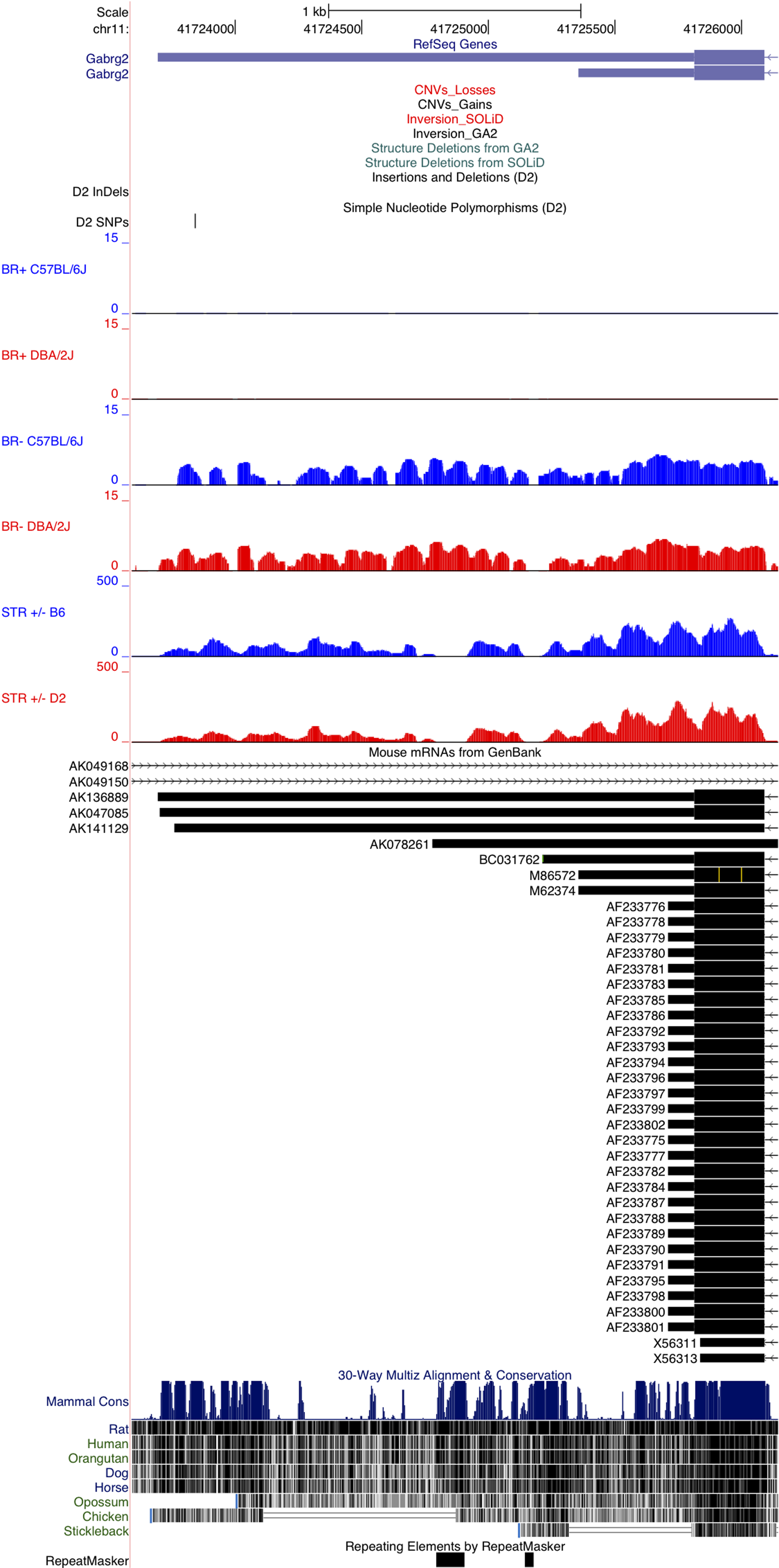

Supplement: Figure S7 — Summary of Gabrg2 3′ UTR expression as assayed using RNA-seq. Data available at ucscbrowser.genenetwork.org. Gabrg2 is located on the (−) strand. Tapering of reads from the proximal to more distal 3′ UTR indicates expression of longer and shorter 3′ UTR isoforms. This is especially prevalent in the striatum, which included only polyadenylated transcripts for RNA-seq. Track 1 shows the chromosomal location at top followed by gene model based on RefSeq as track 2. Larger width indicates coding exons and smaller width indicates 3′ UTR. All known variants between the B6 and D2 genome are indicated as part of track 3. Tracks 4 through 7 summarize the normalized (+) and (−) strand reads for the B6 (blue) and D2 (red) parental strains in whole brain (RiboMinus method). Tracks 8 and 9 show normalized reads in the striatum of 10 B6 and 11 D2 mice (PolyA enrichment method, [75]). For tracks 4 through 9 the scale to the left shows the read number, which will be lower for whole brain since it represents only a single RNA-seq run from one animal. Track 10 shows previously characterized mRNA species in mouse. Track 11 shows the degree of sequence conservation in mammals and Track 12 shows the presence of any repetitive DNA, which is masked by most RNA-seq alignment algorithms. (TIF) [file pone.0034586.s007.tif]
